# Sr(II) and Ba(II) Alkaline Earth Metal–Organic Frameworks (AE-MOFs) for Selective Gas Adsorption, Energy Storage, and Environmental Application

**DOI:** 10.3390/nano13020234

**Published:** 2023-01-04

**Authors:** Nikolas Király, Dominika Capková, Róbert Gyepes, Nikola Vargová, Tomáš Kazda, Jozef Bednarčík, Daria Yudina, Tomáš Zelenka, Pavel Čudek, Vladimír Zeleňák, Anshu Sharma, Vera Meynen, Virginie Hornebecq, Andrea Straková Fedorková, Miroslav Almáši

**Affiliations:** 1 Department of Inorganic Chemistry, Faculty of Science, Pavol Jozef Šafárik University in Košice, Moyzesova 11, SK-041 54 Košice, Slovakia; 2 Department of Physical Chemistry, Faculty of Sciences, Pavol Jozef Šafárik University in Košice, Moyzesova 11, SK-041 54 Košice, Slovakia; 3 Department of Inorganic Chemistry, Faculty of Science, Charles University, Albertov 8, CZ-128 43 Prague, Czech Republic; 4 Department of Electrical and Electronic Technology, Faculty of Electrical Engineering and Communication, Brno University of Technology, Technická 10, CZ-616 00 Brno, Czech Republic; 5 Department of Physics, Faculty of Science, Pavol Jozef Šafárik University in Košice, Park Angelinum 9, SK-041 01 Košice, Slovakia; 6 Institute of Experimental Physics, Slovak Academy of Sciences, Watsonova 47, SK-040 01 Košice, Slovakia; 7 Department of Chemistry, Faculty of Science, University of Ostrava, 30. Dubna 22, CZ-702 00 Ostrava, Czech Republic; 8 Department of Physics, School of Engineering & Technology, Central University of Haryana, Mahendergarh 123031, India; 9 Laboratory of Adsorption and Catalysis, University of Antwerp, Universiteitsplein 1, B-2610 Wilrijk, Belgium; 10 Centre National de la Recherche Scientifique (CNRS), Matériaux Divisé, Interfaces, Réactivité, Electrochimie (MADIREL), Centre de Saint Jérôme, Aix-Marseille University, Avenue Escadrille-Normandie-Niemen, F-133 97 Marseille, France

**Keywords:** metal–organic frameworks, alkaline earth metals, gas adsorption/separation, lithium-sulphur battery, energy storage

## Abstract

Two new alkaline earth metal–organic frameworks (AE-MOFs) containing Sr(II) (**UPJS-15**) or Ba(II) (**UPJS-16**) cations and extended tetrahedral linker (MTA) were synthesized and characterized in detail (UPJS stands for University of Pavol Jozef Safarik). Single-crystal X-ray analysis (SC-XRD) revealed that the materials are isostructural and, in their frameworks, one-dimensional channels are present with the size of ~11 × 10 Å^2^. The activation process of the compounds was studied by the combination of in situ heating infrared spectroscopy (IR), thermal analysis (TA) and in situ high-energy powder X-ray diffraction (HE-PXRD), which confirmed the stability of compounds after desolvation. The prepared compounds were investigated as adsorbents of different gases (Ar, N_2_, CO_2_, and H_2_). Nitrogen and argon adsorption measurements showed that **UPJS-15** has *S_BET_* area of 1321 m^2^ g^−1^ (Ar) / 1250 m^2^ g^−1^ (N_2_), and **UPJS-16** does not adsorb mentioned gases. From the environmental application, the materials were studied as CO_2_ adsorbents, and both compounds adsorb CO_2_ with a maximum capacity of 22.4 wt.% @ 0 °C; 14.7 wt.% @ 20 °C and 101 kPa for **UPJS-15** and 11.5 wt.% @ 0°C; 8.4 wt.% @ 20 °C and 101 kPa for **UPJS-16**. According to IAST calculations, **UPJS-16** shows high selectivity (50 for CO_2_/N_2_ 10:90 mixture and 455 for CO_2_/N_2_ 50:50 mixture) and can be applied as CO_2_ adsorbent from the atmosphere even at low pressures. The increased affinity of materials for CO_2_ was also studied by DFT modelling, which revealed that the primary adsorption sites are coordinatively unsaturated sites on metal ions, azo bonds, and phenyl rings within the MTA linker. Regarding energy storage, the materials were studied as hydrogen adsorbents, but the materials showed low H_2_ adsorption properties: 0.19 wt.% for **UPJS-15** and 0.04 wt.% for **UPJS-16** @ −196 °C and 101 kPa. The enhanced CO_2_/H_2_ selectivity could be used to scavenge carbon dioxide from hydrogen in WGS and DSR reactions. The second method of applying samples in the area of energy storage was the use of **UPJS-15** as an additive in a lithium-sulfur battery. Cyclic performance at a cycling rate of 0.2 C showed an initial discharge capacity of 337 mAh g^−1^, which decreased smoothly to 235 mAh g^−1^ after 100 charge/discharge cycles.

## 1. Introduction

In the last two decades, the study of metal–organic frameworks (MOFs) was one of the fastest-growing areas in chemistry due to their great potential for many applications and structural diversity. MOFs are constructed by linking organic and inorganic building blocks through coordination bonds to form 3D porous polymeric frameworks. The inorganic units can be metal cations or clusters; the latter is widely described as secondary building units (SBUs) with different shapes and sizes. Because of the extraordinary variability degree of building blocks, their enormous surface areas, and the multiple ways of surface functionalization and modification, MOFs have considerable potential in many application areas, such as gas storage [1,2,3,4] and separation [5,6], heterogeneous catalysis [7,8,9,10], drug delivery [11,12,13], water [14,15,16,17] and sensor technology [18,19], magnetic refrigeration [20,21,22], or conductive matrix for sulphur in lithium-sulphur (Li-S) batteries [23,24,25,26].

One of the subgroups of MOF materials is alkaline earth metal–organic frameworks (AE-MOFs), which are constructed from the metal ions of the second group in the periodic table. AE-MOFs are predominantly studied as gas adsorbents and separators for different gases. Carbon dioxide is one of the main greenhouse gases causing global warming. At present, there is an urgent need to develop novel and efficient adsorbents that will selectively adsorb or separate CO_2_ from industrial emissions under atmospheric conditions. The increased selectivity of the material for CO_2_ can be achieved either by introducing specific functional groups -NH_2_, -OH, and -COOH within the linker, by coordinatively unsaturated sites (CUSs), or by post-synthetic modification with amines [27] on the central atoms [28,29,30,31]. CUSs are formed in material activation when coordinated solvent molecules are removed from the coordination sphere of the central atom, and free orbitals (Lewis bases) are formed to serve as primary adsorption sites for CO_2_ [32]. The compound that showed promising results in CO_2_ adsorption is Mg (dobdc), also known as Mg-MOF-74 (dobcd = 2,5-dioxido-1,4-benzenedicarboxylate). It contains 1D honeycomb channels with CUSs lines in the corners of the hexagon. The storage capacity of Mg-MOF-74 is up to 35.2 wt.% CO_2_ @ 1 atm and 23.6 wt.% @ low pressures (0.1 atm) and 23 °C. Mentioned values are higher than the ones obtained for isostructural analogues of M-MOF-74 (M = Zn, Co or Ni) and this observation can be explained by the weight effect of central atoms, the increased affinity of Mg(II) ions for CO_2_ and CUSs [33]. Post-synthetic modification of CUSs with amines is another method used to increase the sorption capacity of the material. As an example, Mg-MOF-74 modified with diamine (diaminopropane, *dap*) and tetraamine (spermine, *spe*) [34,35] (Mg-MOF-74-*dap*) can adsorb 9.1 wt.% CO_2_ from simulated coal flue gas @ 40 °C and 1 bar [34]. The tetraamine-modified material displays 9.7 wt.% CO_2_ capture from simulated natural gas flue streams (4% CO_2_ in N_2_) in the presence of water (2.6%) @ 100°C and atmospheric pressure [35].

Another application is the development of efficient and safe hydrogen storage systems, which is a key parameter to the further implementation of H_2_ in fuel cell technologies, particularly in transport, which is currently the primary producer of greenhouse gases. Among the various approaches to enhancing such systems’ performance, hydrogen adsorption storage based on porous materials is considered a long-term solution due to its excellent ability to store hydrogen under low pressures, good kinetics, and reversibility. Metal–organic frameworks attracted much attention as porous materials for hydrogen storage in the transition ranging from laboratory to commercial applications [36,37,38]. Among the results published to date for the AE-MOFs, UPJS-7 containing Ca(II) ions and MTA (MTA = methanetetrabenzoate) linker with an H_2_ capacity of 3.65 wt.% @ 1 atm and −196 °C [39] shows the best results at low pressures, and the leader compound at high pressure is Be_12_(OH)_12_(BTB)_4_ (BTB = benzene-1,3,5-tribenzoate) with a hydrogen uptake of 9.2 wt.% @ 100 bar, 6.0 wt.% @ 20 bar, and −196 °C or 2.3 wt.% @ 95 bar and RT [40].

A third application, from a technological standpoint, concerns the crucial separation of ethene from ethane in the production of polymers. An example of an efficient separator is UTSA-280, which consists of Ca(II) ions and squaric acid. The mentioned AE-MOF can efficiently separate C_2_H_4_ from C_2_H_6_ due to the shape selectivity of pores (pore cross-sectional area of 14.4 Å^2^), whose size is only suitable for the passage of C_2_H_4_ molecules: the minimum cross-sectional areas of ethene and ethane are 13.7 and 15.5 Å^2^, respectively [41]. Another example is the compound [Ba_2_(BTC)(NO_3_)(DMF)] (BTC = benzene-1,3,5-tricarboxylate, DMF = *N*,*N’*-dimethylformamide), which is able to completely separate methane from the CO_2_/CH_4_ mixture [42].

Finally, the research on energy storage technologies attracted great attention due to the increasing demand for effective portable power sources for mobile electronic devices, electric vehicles, or space missions [43,44]. Lithium-sulphur (Li-S) batteries are considered as one of the most promising candidates to replace currently used lithium-ion batteries, thanks to their enormous energy density (~2600 Wh kg^−1^) and theoretical capacity (1675 mAh g^−1^) [45,46]. Applying AE-MOFs as supports for sulphur in Li-S batteries may suppress their shortcomings due to their unique structure and properties [25,26,47,48,49]. Indeed, during discharging of the Li-S battery, sulphur rebounds with lithium ions: the intermediate products are soluble long-chain polysulphides (Li_2_S*_x_*, 4 ≤ *x* ≤ 8) and the discharge products are insoluble short-chain polysulphides (Li_2_S_2_/Li_2_S) [50,51]. This migration/shuttle of soluble polysulphides leads to poor cycle stability, low coulombic efficiency, and a high self-discharge rate. Capturing sulphur into MOF’s pores may inhibit the polysulphide shuttle during cycling. Furthermore, the insulating characteristic of sulphur can be solved by incorporating sulphur into conductive and porous materials. The MOF-derived carbon materials containing heteroatoms and integrating metal species as a sulphur host can effectively elevate the cycle performance and conductivity of the cathode material [52,53,54].

AE-MOFs are also intensively studied for their high proton conductivity, which is an important attribute for application in fuel cells and biosystems, supercapacitors, batteries, as well as electrochromic and sensor devices [55,56,57,58,59]. AE-MOFs also find application as heterogeneous catalysts in Knoevenagel condensation, Michael addition, Aldol condensation, and hydrogenation of alkenes [60,61,62,63]. Some materials also exhibit high active second harmonic generation (SHG) properties, which are desirable in the areas of IT, lasers, and optoelectronic technologies [64,65]. Other applications to mention may be biomedical applications, such as targeted drug delivery, osteoporosis treatment, biomineralization, and SARS-CoV-2 detection and elimination [66,67,68,69].

Herein, we demonstrate a successful preparation and implementation of two novel MOFs containing alkaline earth metals: Sr(II) **UPJS-15** and Ba(II) **UPJS-16** cations for selective gas adsorption and as a matrix in Li-S batteries (UPJS stands for University of Pavol Jozef Safarik). Both compounds represent a highly porous three-dimensional (3D) framework with a one-dimensional (1D) channel pore system containing extended tetracarboxylic acid (H_4_MTA) based on tetraphenylmethane moiety. After the detailed characterization of the prepared porous materials, the adsorption properties of different gases (Ar, N_2_, CO_2_, and H_2_) were studied. Moreover, the electrochemical properties of activated **UPJS-15** were investigated as a sulphur host in cathode material for Li-S batteries.

## 2. Experimental

### 2.1. Synthetic Part

#### 2.1.1. Used Chemicals

**Figure 1:**
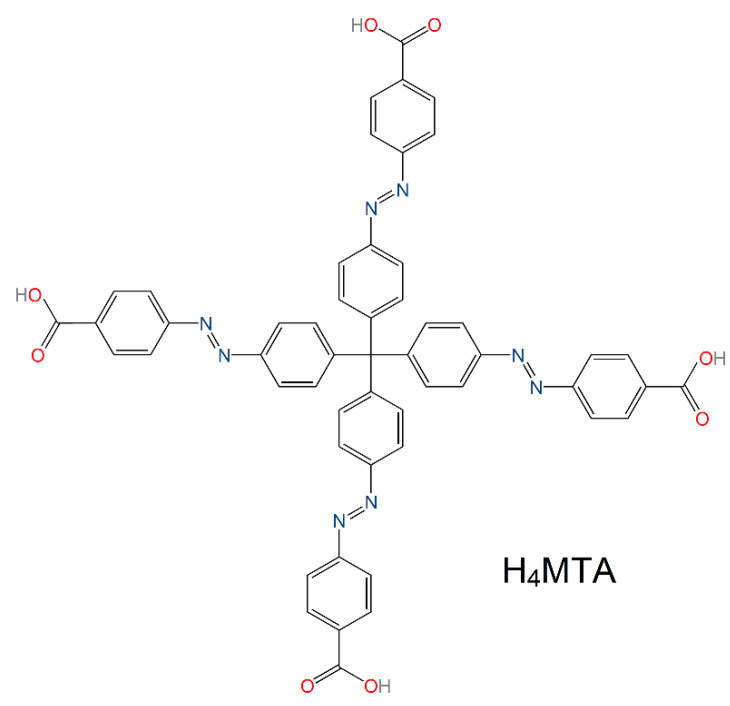
Molecular structure of tetraazo-tetracarboxylic acid (H_4_MTA) linker applied in the preparation of **UPJS-15** and **UPJS-16** materials.

All the chemicals used in the preparation of methanetetrayltetrakis(benzene-4,1diyl)tetrakis(aza))tetrakis(methan-1-yl-1-yliden)tetrabenzoic acid, H_4_MTA (see Figure 1), **UPJS-15**, and **UPJS-16** were purchased from eMolecules, Acros Organics and Sigma-Aldrich companies, and used without further purification. H_4_MTA, as an organic part of MOFs, was synthesized by multi-step organic reactions according to the published literature procedure [70].

#### 2.1.2. Synthesis of UPJS-15 and UPJS-16

Syntheses of {[Sr_2_(MTA)(H_2_O)]·H_2_O·4DMF}_n_ (**UPJS-15 (AS: As-Synthetized)**) and {[Ba_2_(MTA)(H_2_O)]·H_2_O·4DMF}_n_ (**UPJS-16 (AS)**): Orange needle-shaped crystals of the compounds were prepared by hydrothermal synthesis. A mixture of Sr(NO_3_)_2_·4H_2_O (18.5 mg, 0.088 mmol) or Ba(NO_3_)_2_ (22.9 mg, 0.088 mmol) and H_4_MTA (10 mg, 0.011 mmol) and 6 mL of DMF was sealed into a 23 mL glass vial. After the complete dissolution of the reactants, 2 mL of water was slowly added dropwise to the reaction mixture. The vessels were heated to 80 °C at a heating rate of 10 °C min^−1^, held at this temperature for 96 h, and then cooled to ambient temperature. The obtained crystals of materials were filtered, washed several times with DMF, acetone, and dried in the air stream. The yield of as-synthesized samples was 11.3 mg for **UPJS-15 (AS)** and 10.6 mg for **UPJS-16 (AS)**, corresponding to 73% and 69%, respectively, based on H_4_MTA.

Elemental analysis (EA) for **UPJS-15 (AS)** {[Sr_2_(MTA)(H_2_O)]·H_2_O·4DMF}_n_; *M_w_* = 1412.52 g mol^−1^): CHN clcd.: C 55.27%, H 4.57%, and N 11.90%; exp.: C 54.94%, H 4.66%, and N 11.99%.

Elemental analysis (EA) for **UPJS-16 (AS)** {[Ba_2_(MTA)(H_2_O)]·H_2_O·4DMF}_n_; *M_w_* = 1511.93 g mol^−1^): CHN clcd.: C 51.64%, H 4.27%, and N 11.12%; exp.: C 51.44%, H 4.24%, and N 11.37%.

#### 2.1.3. Solvent Exchange Process and Activation of the Compounds

To prepare the materials for gas adsorption measurements, an ethanol exchange process to replace the solvents located in the pores of materials (DMF, H_2_O) was performed. The compounds were prepared by soaking the as-synthesized material in a 98% ethanol solution for two weeks. Then, the suspension was decanted each 24 h. The ethanol-exchanged materials prepared by the above-described procedure were designed as **UPJS-15 (EX = EXchanged)** and **UPJS-16 (EX)** in the following text. Before the gas adsorption measurements, the solvent-exchanged samples were outgassed in a vacuum at 120 °C for 16 h to eliminate solvents from the channel system. The materials were heated gradually at a heating rate of 3 °C min^−1^ with an isothermal step at 60 °C with a soaking time of half hour. Activated materials were designed as **UPJS-15 (AC: ACtivated)** and **UPJS-16 (AC)** in the further text.

#### 2.1.4. Preparation of S-MOF-Based Electrode Material

All chemicals used to prepare electrode slurry and electrolytes were purchased from Timcal or Sigma-Aldrich companies.

Synthesized and activated metal–organic framework **UPJS-15 (AC)** was applied as a carrier for sulphur in Li-S batteries. **UPJS-15 (AC)**, sulphur, and carbon Super P were milled by wet ball milling in ethanol at 500 rpm for 30 min using a zirconium oxide grinding jar. The electrode tar was synthesized by combining sulphur, **UPJS-15 (AC)**, Super P, and poly (vinylidene) difluoride (PVDF) in *N*-methyl-2-pyrrolidone (NMP), and the final mass ratio of electrode components was 60:15:15:10. The cathode slurry was overlayed on aluminium foil using carbon modification on the surface by a coating bar and subsequently dried in the oven at 60 °C for one day. Electrode disks with a diameter of 18 mm were compressed with a pressure of 350 kg cm^−2^ and then dried under a vacuum at 60 °C inside a glove box. The sulphur loading in the cathode material was controlled at around 2.8 mg cm^−2^.

#### 2.1.5. Cell Assembly

The Li-S cells were constructed in a glove box filled with high-purity argon (Jacomex) to El-Cell^®^ by using a Li metal foil as an anode, **S/UPJS-15** as a cathode, and a glass fibre-based separator. The electrolyte was in a composition of 0.7 M solution of lithium bis(tri-fluoromethanesulfonyl) imide (LiTFSI) and 0.25 M solution of LiNO_3_ in 1,3-dioxolane (DOL) and 1,2-dimethoxyethane (DME) with the volume ratio of 1:2.

### 2.2. Methods and Characterization

#### 2.2.1. Elemental and ICP-MS Analyses

Elemental analysis (EA) was carried out using a CHNOS Elemental Analyzer Vario MICRO from Elementar Analysensysteme GmbH with ~5 mg mass.

Inductively coupled plasma-mass spectrometry (ICP-MS) analysis was applied to determine the strontium (II) and barium (II) quantity in the as-synthesized compounds. Prior to the measurement, the materials were mineralized in a warm aqua regia.

#### 2.2.2. Infrared Spectroscopy

The infrared spectra of the materials were performed at room temperature and recorded on an Avatar FTIR 6700 apparatus in the wavenumber range of 4000–400 cm^−1^ with 64 repetitions for one spectrum and a resolution of 4 cm^−1^ using the KBr pellets. Samples were assembled in the form of KBr discs with a weight ratio (sample: KBr/1: 100). Before infrared measurements, potassium bromide was heated at 600 °C for 16 h and further cooled in a desiccator. Diffuse reflectance infrared Fourier transform (DRIFT) measurements were carried out using the same device equipped with a DTGS detector in the wavenumber region of 4000–500 cm^−1^. Each given spectrum averages 200 measurements, while the resolution is 4 cm^−1^. The Praying Mantis in situ heating cell under vacuum was used for the measurements, and as a reference background, the pure KBr was used.

#### 2.2.3. Thermal Analysis

The thermal properties of as-synthesized, solvent-exchanged, and activated materials were investigated by thermogravimetric analysis (TA) using platinum crucibles and a sample weight of ~25 mg. TA measurements were carried out on a TGA Q500 apparatus. The materials were heated in a dynamic atmosphere of air with a flow rate of 60 cm^3^ min^−1^. The samples were measured in the temperature range of 25–800 °C with a heating rate of 10 °C min^−1^.

#### 2.2.4. High Energy Powder X-ray Diffraction (HE-PXRD) with In Situ Heating and Powder X-ray Diffraction (PXRD)

In situ heating high-energy powder X-ray diffraction (HE-PXRD) measurements were performed at the P02.1 beamline of the PETRA III storage ring in Deutsches Elektronen Synchrotron (DESY, Hamburg, Germany). The beam size during experiments was set to 0.6 × 0.6 mm^2^, and the radiation wavelength was *λ* = 0.20727 Å. The samples were added in a glass capillary with a wall thickness of 20 μm and a diameter of 1.2 mm. Diffracted photons from the samples were recorded using a Perkin Elmer PEI1621 2D detector. Sample to detector distance (1526 mm) and detector tilt to beam axis were calibrated and calculated using a diffraction pattern from LaB_6_ as standard reference material. The compounds were heated in the temperature range of 30–700 °C with a heating rate of 5 °C min^−1^, while the diffraction data were recorded every 50 °C. The 2D diffraction patterns obtained were integrated using the Fit2D program [71].

Powder X-ray diffraction (PXRD) measurements were performed in the reflection geometry using a Rigaku Ultima IV multipurpose diffractometer. To obtain a parallel and pure X-ray beam for PXRD measurements, the initially divergent Cu/Kα radiation (*λ* = 1.54056 Å) emitted from the X-ray lamp was further guided through a multilayer mirror and a set of slits. PXRD experiments were conducted by 2*θ* continuous scans from 5° to 60° with a measurement speed of 0.5° min^−1^, and a NaI scintillation detector was used for the recording of diffracted photons.

#### 2.2.5. Scanning Electron Microscopy and Energy-Dispersive X-ray Spectroscopy

The morphology of the materials for electrochemical measurements was studied on a TESCAN VEGA3 equipped with an EDAX analyser using energy-dispersive X-ray spectroscopy (EDS) and scanning electron microscopy (SEM).

#### 2.2.6. Single-Crystal X-ray Structure Measurements and Determination

The single-crystal X-ray diffraction (SC-XRD) data set was collected on a Nonius Kappa CCD diffractometer equipped with a Bruker APEX II detector. For **UPJS-15 (AS)** and **UPJS-16 (AS)** Cu/Kα (*λ* = 1.54178 Å) @ −153(2) °C, and data reduction was conducted using the diffractometer software. The phase problem was solved by direct methods and refined with full-matrix least-squares on *F*^2^ using the Shelxl-2018 program suite [72]. All atoms, excluding hydrogen atoms, were refined anisotropically. Aromatic hydrogen atoms were included in an ideal position, with *U_iso_(H)* assigned to 1.2*U_eq_* of the adjacent carbon atom and the C−H bond fixed to 0.95 Å. Guest molecules located in the cavities of the materials were subtracted by the SQUEEZE procedure in Platon [73]. The structure images were visualized using DIAMOND software [74]. Crystal data for **UPJS-15 (AS)** and **UPJS-16 (AS)** are listed in Table S1 in Supplementary Materials and selected bond lengths and angles in Tables S2 and S3 in Supplementary Materials. The crystal structures of **UPJS-15 (AS)** and **UPJS-16 (AS)** under the registration numbers 2117419 and 2117420, respectively, were deposited in the Cambridge Crystallographic Data Centre (CCDC) and can be retrieved free of charge from https://www.ccdc.cam.ac.uk/structures (accessed on 4.1.2023). The topological analysis of the frameworks was performed using ToposPro software [75,76].

#### 2.2.7. Gas Adsorption

Prior to the gas physisorption experiments, **UPJS-15 (AC)** and **UPJS-16 (AC)** samples were degassed under a vacuum for 16 h @ 120 °C.

Nitrogen (99.999% purity) adsorption experiments were measured on an ASAP 2020 Micromeritics apparatus @ −196 °C, and argon (99.999%) adsorption measurements @ −186 °C on a Quantachrome AUTOSORB-1-MP automated gas sorption system. Based on the nitrogen and argon adsorption/desorption measurements, the BET specific surface area (*S_BET_*) of each sample was calculated by adsorption data in a *p/p*_0_ range from 0.03 to 0.15. DFT method was used for the calculation of the pore size diameter (*d*) and pore volume (*V_p_*).

CO_2_ adsorption isotherms (99.999% purity) @ 0 °C and 20 °C were carried out using a Quantachrome AUTOSORB-iQ-C with a combined dynamic and volumetric sorption system. The CO_2_ isotherms were performed in the range of 0.5–101 kPa of abs. pressure.

The H_2_ (99.999% purity) adsorption measurements were performed on the Autosorb iQ-XR static manometric adsorption system developed by Quantachrome Instruments. The hydrogen adsorption isotherms were collected @ −196 °C in the range of 0.5–101 kPa of absolute pressure.

#### 2.2.8. DFT Modelling and Computational Studies

Theoretical studies were based on density functional theory (DFT) computations using Gaussian16, Revision C.01 [77]. All computations utilized the B3LYP functional [78,79,80], the Stuttgart/Dresden ECP [81] for the Sr atoms, and the 6-311+G(3d,p) basis set for all other atoms. The correction for empirical dispersion was included as Grimme’s dispersion using the D3 damping function [82].

MTA linker: The geometry of MTA molecule was first fully optimized by imposing the *S_4_* point group for this anionic species. Since the optimization could only utilize *C*_2_ symmetry as the largest Abelian subgroup of *S*_4_, the density matrix was symmetrized before each SCF cycle to match the molecular symmetry. The geometry optimization procedure used a Hessian estimated prior the first optimization step and was updated at each step during the course of optimization. The three MTA–CO_2_ models were obtained by performing partial optimizations on models, which included the optimized geometry of a frozen MTA linker, while complete optimization was allowed for the auxiliary CO_2_ molecules placed into three different starting positions. After obtaining appropriate stationary points on the potential hypersurfaces, the total interaction energies between MTA linker and the CO_2_ molecules were obtained from counterpoise computations. These counterpoise computations subdivided models into two fragments—MTA linker and CO_2_; their interaction energy was then obtained as the difference of the full-system total energy and the sum of the total energies of the two fragments using the full-system basis set for both fragments.

Sr(II) cluster: The geometry optimization of the Sr(II) cluster was initially attempted too, but due to termination effects, the structure motif underwent major alterations upon optimization and got into a significant disagreement with the experimental motif (mostly in the orientation of the organic residues). These undesired geometry changes could not be fully prevented even upon freezing some redundant coordinates (typically involving the positions of the Sr atoms) during the optimization. The geometry of cluster was further used as obtained from the diffraction experiment and by replacing its diazo groups for hydrogen atoms with the C–H interatomic distances fixed at 0.95 Å and attempting no geometry optimization. The interaction energy between Sr(II) cluster and three CO_2_ molecules was obtained by conducting a partial optimization for cluster–CO_2_ that included the cluster part frozen, while the three CO_2_ molecules were fully free to optimize. After reaching an appropriate stationary point, the interaction energy between the cluster and the CO_2_ molecules was obtained by a counterpoise computation on the optimized model, where the supersystem was divided into two fragments—he cluster (first fragment) and the three CO_2_ molecules (second fragment).

#### 2.2.9. Electrochemical Characterization

The electrochemical measurements were carried out using a VMP3 potentiostat (Biologic). Electrochemical characterization was studied by galvanostatic cycling (GC) and cycling voltammetry (CV). CV was carried out within the potential window from 1.8 to 3.0 V vs Li/Li+ at a scan rate of 0.1 mV s^−1^. GC measurement was realized with the potential range of 1.8–2.8 V vs Li/Li+. The capacities obtained from cycling at 0.2 C were related to the weight of sulphur.

## 3. Results and Discussion

Tetrahedral tetraazo-tetracarboxylic acid (H_4_MTA) was produced by the multistep organic synthesis based on the described procedure [70]. A solvothermal reaction between H_4_MTA and Sr(NO_3_)_2_·4H_2_O or Ba(NO_3_)_2_ in a DMF/H_2_O mixture afforded orange needle-shaped single crystals of {[Sr_2_(MTA)(H_2_O)]·H_2_O·4DMF}_n_ (**UPJS-15 (AS)**) and {[Ba_2_(MTA)(H_2_O)]·2DMF·3H_2_O}_n_ (**UPJS-16 (AS)**) suitable for SC-XRD analysis and further characterization. These formulae were proposed based on the SC-XRD measurements, elemental and ICP-MS analyses, thermogravimetric analysis (TG), and infrared spectroscopy (IR). Thermal stability and activation condition of the compounds were studied by the combination of in situ heating HE-PXRD, in situ heating DRIFT, and TG. The activated compounds were further investigated as gas sorbents for argon, nitrogen, carbon dioxide and hydrogen, and as an additive in Li-S batteries.

### 3.1. Description of Crystal Structures

**Figure 2:**
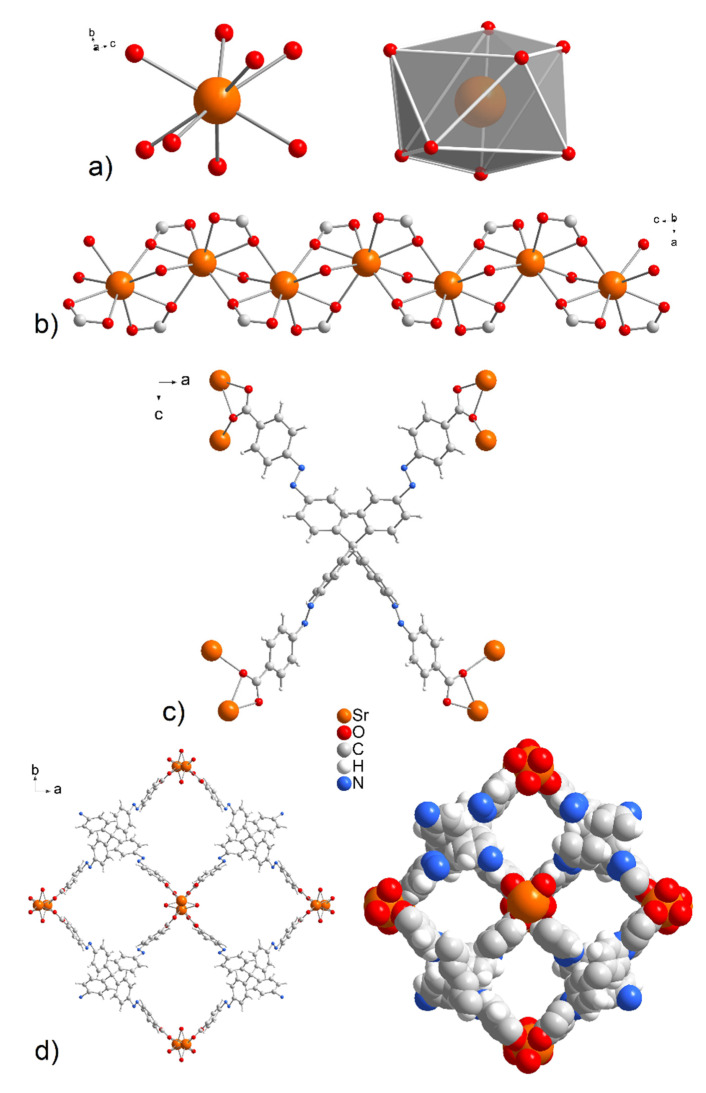
(**a**) The coordination environment and (**b**) zig-zag arrangement of central atoms in the crystal structures of **UPJS-15 (AS)** and **UPJS-16 (AS)**. (**c**) *Chelate-anti* coordination modes of MTA^4−^ linker. (**d**) A view of the 1D tetragonal pores propagating along the *c* crystallographic axis in ball and stick and spacefill modes.

A single-crystal structure determination reveals that **UPJS-15 (AS)** (see Figure 2) and **UPJS-16 (AS)** are isostructural and crystallize in the tetragonal space group *I-42d*, containing four formula units, and the cell lengths *a* = 32.3789(8); 31.6708(9) Å, *c* = 7.3286(2); 7.7689(2) Å, respectively. Detailed information about the crystal structure refinements is listed in Table S1 in Supplementary Materials. Based on the crystallographic review of the CCDC database, it can be stated that the tetragonal system is preferred for compounds containing tetrahedral carboxylate ligands [83]. The asymmetric unit of the compounds contains one quarter of the MTA linker, one water molecule, and one alkaline earth metal ion. All carboxylic groups of H_4_MTA linker are deprotonated and bonded to the central atoms in *chelate-anti* mode. One MTA ion coordinates to eight alkaline earth central atoms, as can be seen from Figure 2c. The tetrahedral MTA linker lost its *C*_3_ rotational axes by distorting the angles of its two shoulders from the ideal 109°28′ to 111.9°. Additionally, phenyl rings that are connected by an azo bond display a dihedral angle of 5.3°, which disrupts ligand rigidity. Based on the above facts, the linker symmetry was reduced from tetrahedral (*T_d_*) to *D_2d_*, resulting in the tetragonal space group of the compounds. The M-O bond distances have typical lengths between 2.499(4) and 2.621(4) Å for Sr-O and 2.676(9)–2.842(11) Å for Ba-O. Selected bond distances and angles in the crystal structure of compounds are summarized in Table S2 for **UPJS-15 (AS)** and Table S3 for **UPJS-16 (AS)** in Supplementary Materials. One coordinated water molecule completed the coordination sphere of each central atom, which makes a bridge between two alkaline metal ions. According to the described coordination of ligands, the donor set of each central ion is [MO_8_], and the coordination polyhedron can be expressed as a distorted tetragonal antiprism (see Figure 2a). By bridging the carboxylate and aqua ligands, the central atoms are arranged in 1D linear chains propagating along the *c* crystallographic axis (see Figure 2b). The distances between M···M within the polymeric chains are 3.805 Å and 4.119 Å for Sr(II) and Ba(II), respectively. The crystal structures of **UPJS-15 (AS)** and **UPJS-16 (AS)** can be described as 3D polymeric frameworks, which exhibit [6,8-c] network topology according to ToposPro analysis [75,76]. In the frameworks, a 1D channel system along the *c* crystallographic axis is present, and the sizes of channel apertures are 11.19 × 10.10 Å^2^ for **UPJS-15 (AS)** and 10.69 × 9.70 Å^2^ for **UPJS-16 (AS)** (see Figure 2d). The total accessible volume in desolvated **UPJS-15 (AS)** and **UPJS-16 (AS)**, after removal of guest solvents using squeeze procedure, was calculated using the Platon/Void procedure [73] to be 3673.6 Å^3^ and 3103.5 Å^3^, representing 47.8% and 39.8%, respectively.

### 3.2. Characterization, Thermal Stability and Activation Study

**Figure 3:**
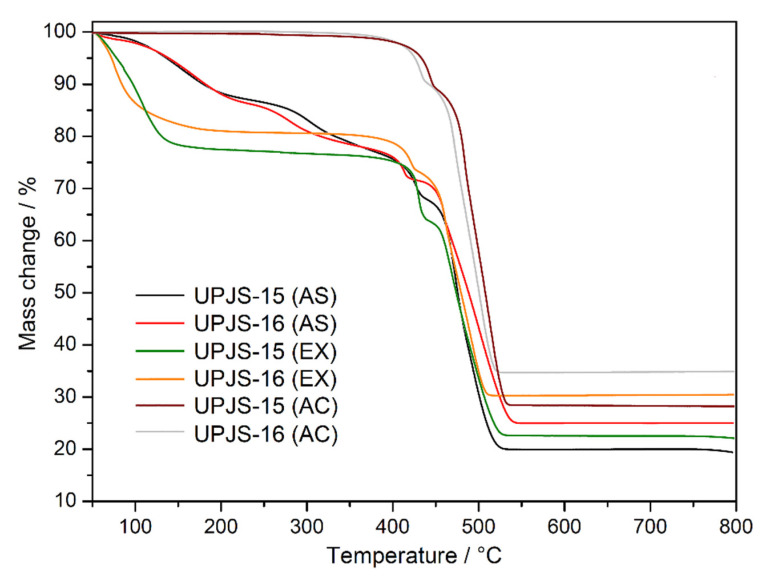
Thermogravimetric curves of as-synthesized (AS), solvent-exchanged (EX), and activated (AC) **UPJS-15** and **UPJS-16** samples in the air atmosphere.

The thermal stability and activation of the prepared compounds were initially studied by thermal analysis in an air atmosphere in the [30–800 °C] temperature range; the measured TG curves are shown in Figure 3. The desolvation process of as-synthesized compounds **UPJS-15 (AS)** and **UPJS-16 (AS)** (see black and red curves in Figure 3) occurs in the [30–380 °C] temperature range in two decomposition steps. The total weight loss in the mentioned temperature range is 22.98 wt.% for **UPJS-15 (AS)** and 22.04 wt.% for **UPJS-16 (AS)**, and corresponds to the release of 4 DMF and 2 H_2_O (clcd. mass change 23.25 wt.% for **UPJS-15 (AS)** and 21.72 wt.% for **UPJS-16 (AS)**. Thermal decomposition of the polymeric framework occurs, by further sample heating in the [380–550 °C] temperature range, also in two decomposition steps. The observed weight losses of 56.90 wt.% and 51.73 wt.% are in good agreement with the calculated values of 55.85 wt.% and 52.18 wt.% for **UPJS-15 (AS)** and **UPJS-16 (AS)**, respectively. Based on PXRD measurements (see Figure S1 in Supplementary Materials), the resulting thermal decomposition products are SrCO_3_ and BaCO_3_ [84,85]. The experimental residual masses correlate well with calculated values (exp. 20.12 wt.%, clcd. 20.90 wt.% for SrCO_3_; exp. 26.23 wt.%, clcd. 26.10 wt.% for BaCO_3_). In ethanol-exchanged samples, the solvent molecules are released at lower temperatures compared to as-synthesized materials. The desolvation of **UPJS-15 (EX)** and **UPJS-16 (EX)** (see green and orange curves in Figure 3) occurs in the temperature range of [30–150 °C]. Subsequently, the desolvated compounds are thermally stable up to 360 °C, and, as seen by the plateau on TG curves, further heating of the materials leads to their thermal decomposition. Successful activation of the compounds is evident from the obtained TG curves of **UPJS-15 (AC)** and **UPJS-16 (AC)** (see wine and grey curves in Figure 3) because no significant weight loss is observed up to 400 °C.

**Figure 4:**
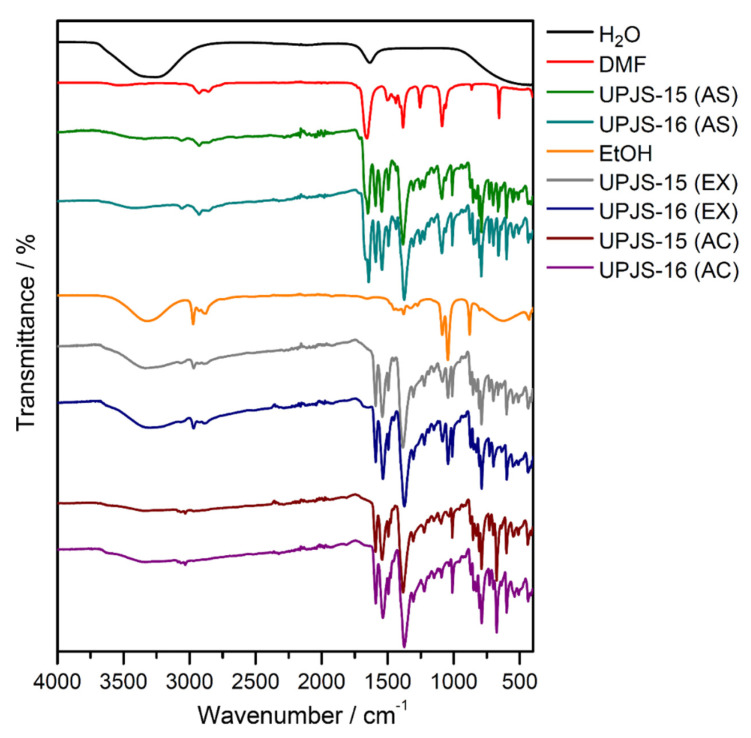
IR spectra of as-synthesized (AS), ethanol-exchanged (EX), and activated (AC) forms of **UPJS-15** and **UPJS-16** and solvents (H_2_O, DMF, and EtOH).

Infrared spectroscopy was applied to confirm the presence of building blocks and solvents (MTA, DMF, and water) in the materials and also to monitor the solvent exchange and activation processes. The IR spectra of compounds are shown in Figure 4, and the assignment of characteristic absorption bands to the bond type vibrations is summarized in Table 1. The isostructurality of the materials is also shown in infrared spectroscopy by the similarity of obtained IR spectra (see Figure 4). The presence of coordinated and crystallization water molecules in as-synthesized **UPJS-15 (AS)** and **UPJS-16 (AS)** materials is evident from the broad absorption band about 3400 cm^−1^, which could be attributed to the stretching *ν*(OH) vibrations. DMF molecules located in the cavities are evidenced by the stretching vibration of aliphatic *ν*(CH) vibration in the region 3050–3060 cm^−1^ and the strong and sharp absorption band of carbonyl groups (*ν*(C=O)) in the range of 1640–1650 cm^−1^ for both compounds. The unambiguous presence of the mentioned solvents was confirmed by comparing the IR spectra of water and *N*,*N*′-dimethylformamide with the synthesized compounds (see Figure 4). The MTA linker in the compound´s frameworks is evident from the presence of several characteristic absorption bands: aromatic stretching vibrations *ν*(CH)_ar_, *ν*(C=C)_ar_ and carboxylate symmetric and antisymmetric stretching (*ν*(COO^−^)_s_, *ν*(COO^−^)_as_), as well as deformation vibrations (*δ*(COO^-^)) (see Figure 4 and Table 1). The successful ethanol exchange process in the IR spectra of **UPJS-15 (EX)** and **UPJS-16 (EX)** was confirmed by the absence of the *ν*(C=O) band, which is characteristic for DMF, and also by the presence of three new aliphatic CH vibrations in the interval 2966–2844 cm^−1^. All absorption bands typical for MTA were observed in the samples after the solvent exchange process. Figure 4 also shows the IR spectra of activated compounds (AC), which confirm the successful removal of solvents after the activation process due to the absence of characteristic absorption bands for solvents, ethanol, and water (see Table 1).

**Figure 5:**
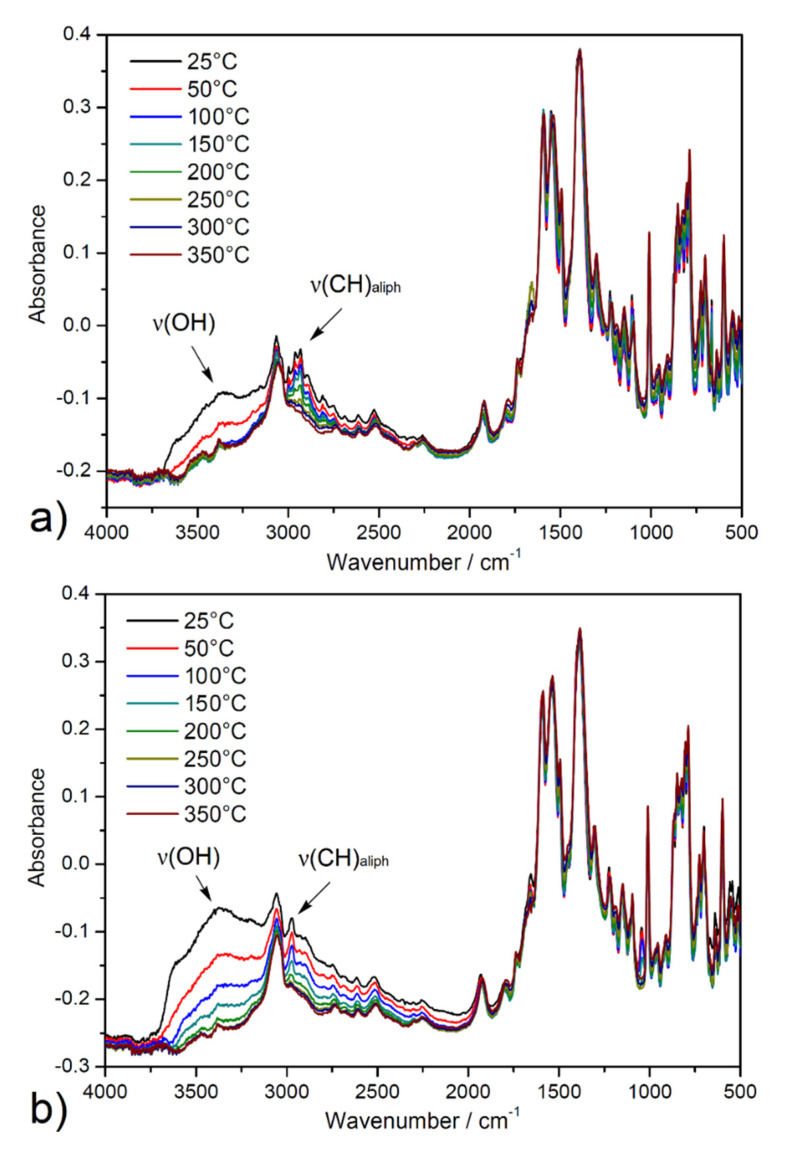
In situ heating DRIFT spectra of solvent-exchanged samples (**a**) **UPJS-15 (EX)** and (**b**) **UPJS-16 (EX)** at selected temperatures (25, 50, 100, 150, 200, 250, 300, and 350 °C) under a vacuum.

The activation process of ethanol-exchanged samples was also investigated by in situ heating infrared spectroscopy (DRIFT) under a vacuum from ambient temperature up to 350 °C (see Figure 5). A broad band dip around 3400 cm^−1^ was observed, corresponding to the O-H stretching vibrations (*ν*(OH)) of crystallization ethanol and coordinated water molecules from the pore system. Moreover, a decreased intensity of the absorption bands under 3000 cm^−1^ to 2800 cm^−1^ was observed that corresponds to the stretching vibrations of aliphatic C-H groups (*ν*(CH)_aliph_) of the aliphatic system of ethanol. Otherwise, the other bands in the spectrum were without changes, confirming the thermal stability of porous materials during the degassing process under a vacuum. The results of the described study are important, especially in the process of material desolvation that occurs during the activation of materials during adsorption measurements.

**Figure 6:**
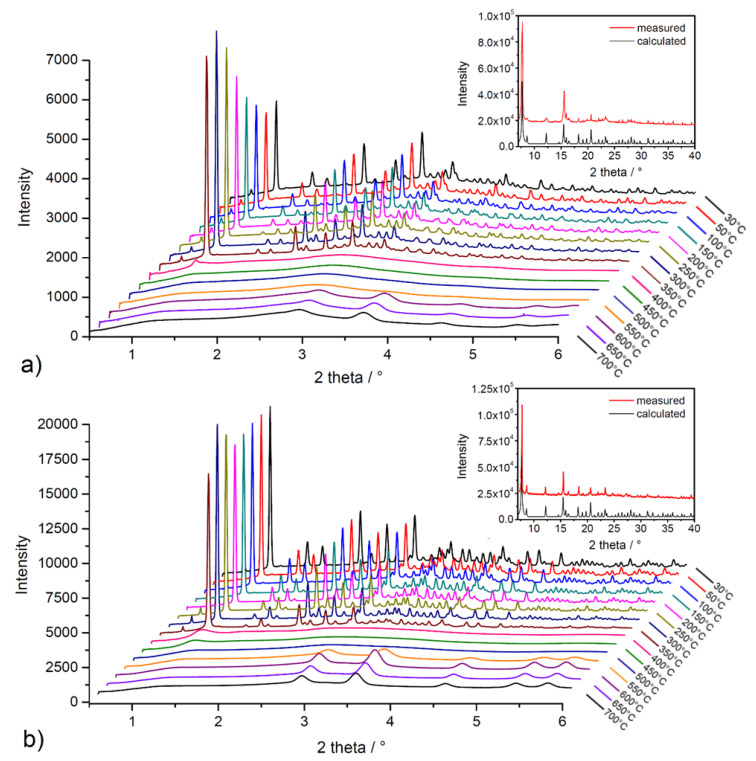
In situ heating HE-PXRD patterns of (**a**) **UPJS-15 (EX)** and (**b**) **UPJS-16 (EX)**. Insets show the comparison of measured and calculated PXRD patterns from SC-XRD data.

High-energy powder X-ray diffraction (HE-PXRD) measurements at DESY (Hamburg, Germany) were performed to investigate the framework stability/lability of solvent-exchanged materials during in situ heating. Figure 6 shows comparisons of measured diffraction patterns with calculated patterns of the compounds from the results of single-crystal X-ray diffraction analysis (SC-XRD) (see insets in Figure 6a,b) and results of in situ heating HE-PXRD measurements in the temperature range of 30–700 °C with 50 °C steps (see Figure 6a,b). As can be seen from the comparison in insets of Figure 6, the measured and calculated diffraction patterns of the materials are in good agreement, representing the phase purity of the bulks and the stability of the compounds during the solvent exchange process. From the results of in situ HE-PXRD measurements (see Figure 6a,b), it can be stated that the materials show a similar, moderate thermal stability, which is in good agreement with the conclusions of TG experiments (see text above). The compounds are stable in the temperature range of 30–350 °C due to the presence of characteristic diffraction peaks for the compounds without the presence of a phase change. According to Kitagawa´s MOF classification, materials **UPJS-15** and **UPJS-16** should be included in the second generation, with stable and robust frameworks without the presence of guest molecules in the channels [86]. Above 350 °C in the temperature range of 400–500 °C, the framework’s collapse is associated with the thermal decomposition of the MTA linker, as only an amorphous phase is present, and no clearly defined diffraction peaks are present in the patterns. In the temperature range of 550–700 °C, the formation of new diffraction peaks can be observed, which correspond to the creation of carbonates of the respective metals as the final decomposition products (see Figure S1 in Supplementary Materials).

### 3.3. Gas Adsorption and DFT Modelling/Calculation

To investigate the porosity, storage capacity, and textural properties of prepared materials, argon (@ −186 °C) and nitrogen (@ −196 °C) adsorption/desorption experiments on ethanol-exchanged samples were performed. Subsequently, adsorption measurements of hydrogen (@ −196 °C) and carbon dioxide at two different temperatures (@ 0 °C and @ 20 °C) were realized. The samples were degassed under a vacuum at 120 °C for 16 h to remove solvents from the channel system. All adsorption measurements were carried out on the same batch of the material, and obtained results from gas adsorption experiments are described below.

**Figure 7:**
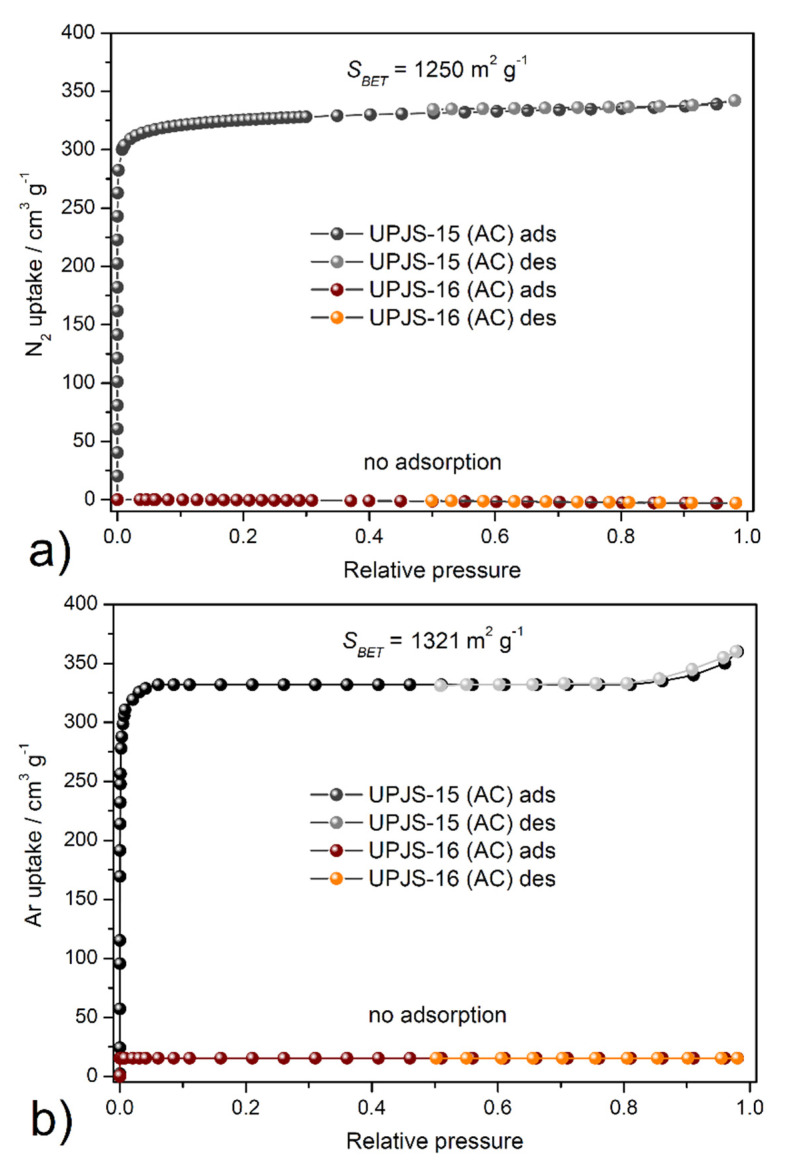
Adsorption/desorption isotherms of (**a**) nitrogen @ −196 °C and (**b**) argon @ −186 °C on **UPJS-15 (AC)** and **UPJS-16 (AC)**.

The textural properties, such as *S_BET_* area, pore volume, and pore size, were investigated by nitrogen and argon adsorption experiments @ −196 °C and −186 °C, respectively. Obtained adsorption isotherms are depicted in Figure 7, and calculated textural parameters are summarized in Table 2. The adsorption isotherms for **UPJS-15 (AC)** (see Figure 7) can be described as *type Ia* by IUPAC classification [87], which is typical for samples with narrow micropores. BET areas (*S*_BET_) of the activated sample **UPJS-15 (AC)** were calculated according to the adsorption data in the *p/p*_0_ range from 0.03 to 0.15. The DFT method was used for the calculation of the pore volume (*V*p) and pore size (*d*). Evaluation of data from nitrogen and argon adsorption analysis of **UPJS-15 (AC)** using the BET equation gave a value of the specific surface area of 1250 m^2^ g^−1^ from the N_2_ adsorption isotherm and 1321 m^2^ g^−1^ from the Ar adsorption isotherm. The micropore volume (*V_p_*) and the average pore size diameter (*d*) of the sample were calculated as 0.83 cm^3^ g^−1^ and 1.76 nm from nitrogen adsorption and 0.85 cm^3^ g^−1^ and 1.82 nm from argon adsorption, respectively.

On the other hand, sample **UPJS-16 (AC)** adsorbs only a limited amount of adsorbates used. According to obtained results, the compound can be described as non-porous, although in both samples, the same channel system, according to results from SC-XRD analysis, was observed. However, it should be noted that **UPJS-16 (AC)** adsorbs carbon dioxide (see text below).

**Figure 8:**
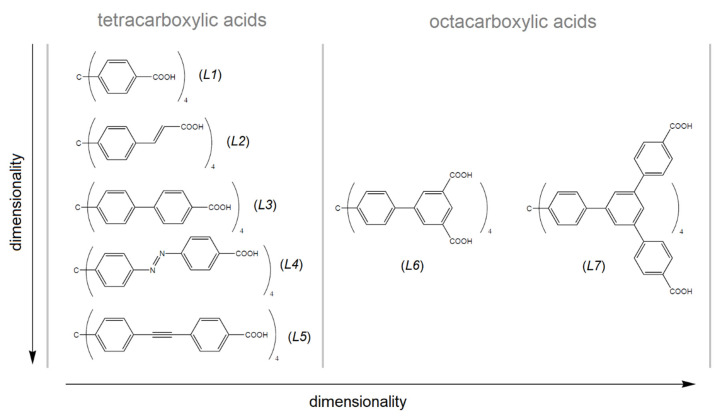
Tetrahedral carboxylate linkers containing tetraphenylmethane core used in the MOF preparation. *L1* = methanetetrabenzoic acid, *L2* = methanetetracinnamic acid, *L3* = 4,4′,4″,4‴-(4,4′,4″,4‴-methanetetrayltetrakis(benzene-4,1-diyl)tetrakis(ethyne-2,1-diyl))tetrabenzoic acid, *L4* = 4,4′,4″,4‴-(4,4′,4″,4‴-methanetetrayltetrakis(benzene-4,1-diyl)tetrakis(aza))tetrakis(methan-1-yl-1-yliden)tetrabenzoic acid, *L5* = 4,4′,4″,4‴-(4,4′,4″,4‴-methanetetrayltetrakis(benzene-4,1-diyl)tetrakis(ethine))tetrakis(methan-1-yl-1-yliden)tetrabenzoic acid, *L6* = 4,4′,4″,4‴-(4,4′,4″,4‴-methanetetrayltetrakis(benzene-4,1-diyl)tetrakis(ethyne-2,1-diyl))isophthalic acid, *L7* = f tetrakis{3,5-bis[(4-carboxyl)phenyl]phenyl}methane.

Table 2 summarizes the textural properties of MOFs made by selected tetrahedral linkers, which consist of a tetraphenylmethane core (see Figure 8) [39,88,89,90,91,92,93,94,95,96,97,98,99,100,101,102,103], which is similar to the presented compounds. As can be seen from Table 2, **UPJS-15 (AC)** has a larger surface area compared to compounds that were prepared from a shorter linker, methanetetrabenzoic acid (linker *L1*). The exception is the compound MOF-812, which contains a robust Zr(IV) cluster with a SBET area of 1390 m^2^ g^−1^, comparable to **UPJS-15 (AC)**. As the arm size bonded to the central methane carbon of the linker increases, the *S_BET_* area of materials also increases. In the case of linker *L3* (see Figure 8), a lower surface area was obtained for Cu(II) [92] and Co(II) [99] compounds, and similar results were observed for materials containing Zn(II) ions [99,100]. An almost three times higher *S_BET_* value was obtained for PCN-521 (3411 m^2^ g^−1^) [101], which is structurally similar to MOF-812, and contains a Zr(IV)-hydroxo cluster. The identical *L4* linker (MTA) was so far used in the synthesis of four MOF compounds, and based on the results summarized in Table 2, **UPJS-15 (AC)** has the largest surface area. For compounds with bulkier and more robust linkers (*L5*, *L6,* and *L7,* see Figure 8), either lower ({[Cu_2_(*L5*)(H_2_O)_2_]·12DEF·26H_2_O}_n_, *S_BET_* = 262 m^2^ g^−1^; {[Cu_2_(H_4_*L7*)(H_2_O)_2_]·18DMF·5H_2_O}_n_, *S_BET_* = 262 m^2^ g^−1^), or two times higher (NOTT-140, *S_BET_* = 2620 m^2^ g^−1^; {[Zn_12_(OH)_4_(H_2_O)_4_(*L7*)(H_2_*L7*)_2_]·G}_n_, *S_BET_* = 2718 m^2^ g^−1^) *S_BET_* values compared to **UPJS-15 (AC)** were published.

**Figure 9:**
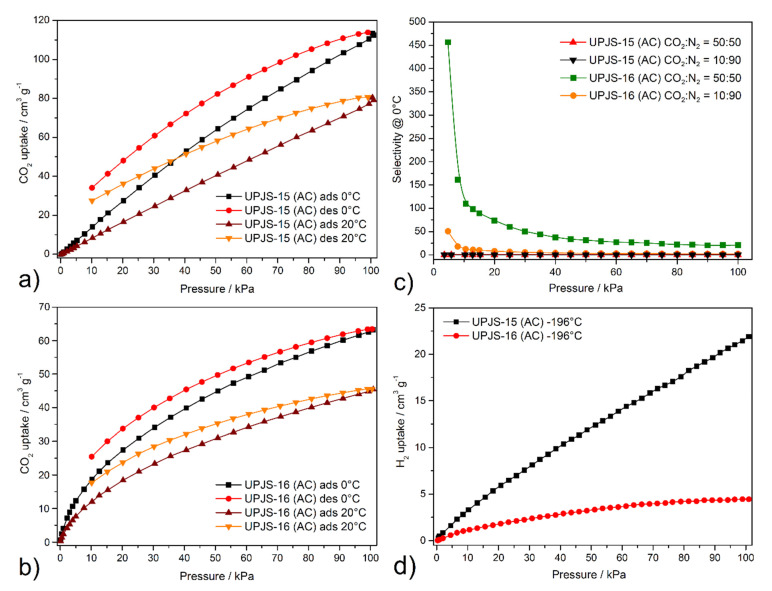
Carbon dioxide adsorption/desorption isotherms of (**a**) **UPJS-15 (AC)** and (**b**) **UPJS-16 (AC)** measured @ 0 and 20 °C. (**c**) IAST selectivities predicted between 5 and 100 kPa for equimolar and 10:90 binary gas mixture of CO_2_/N_2_ on **UPJS-15 (AC)** and **UPJS-16 (AC)** @ 0 °C. (**d**) Hydrogen adsorption isotherms measured @ −196 °C.

The carbon dioxide isotherms were measured on the same batch of samples as previous adsorption measurements, and the same degassing conditions were applied. Carbon dioxide isotherms of compounds **UPJS-15 (AC)** and **UPJS-16 (AC)** measured @ 0 °C and 20 °C are shown in Figure 9. Material **UPJS-15 (AC)** adsorbs CO_2_ up to 113.9 cm^3^ g^−1^ corresponding to 22.4 wt.% (5.08 mmol g^−1^) @ 0 °C and 101 kPa, and 80.8 cm^3^ g^−1^ corresponding to 14.7 wt.% (3.34 mmol g^−1^) @ 20 °C and 101 kPa. The carbon dioxide adsorption measurements for **UPJS-16 (AC)** showed an adsorption capacity of 63.23 cm^3^ g^−1^, corresponding to 11.5 wt.% (2.63 mmol g^−1^) and 45.57 cm^3^ g^−1^ corresponding to 8.4 wt.% (1.89 mmol g^−1^) @ 0 and 20 °C and 101 kPa, respectively.

When comparing the CO_2_ capacities of MOF compounds containing ligands with *T_d_* symmetry (see Table 2) and **UPJS-15 (AC)** material, it could be stated that the obtained results exceed many compounds regardless of the linker size used. Furthermore, it should be noted that **UPJS-15 (AC)** has the highest CO_2_ storage capacity of the mentioned compounds under the same experimental conditions (temperature and pressure). A value of 22.4 wt.% @ 0 °C and 101 kPa is comparable to the 2-aminoterephthalate linker containing compound CUI-1 (24.1 wt.% CO_2_) [104] or SNU-5 (24.1 wt.% CO_2_) [105] and SNU-21 (24.1 wt.% CO_2_) [106], which can be included among the top unmodified MOF CO_2_ adsorbents. The increased values of carbon dioxide storage capacities in the described compounds can be explained by the presence of:

**1. Coordinatively unsaturated sites (CUS)**—one CUS per each central atom in the 1D linear chain within the framework (see description of crystal structure (Section 3.1) above).

**2. MTA linker**—the presence of an azo group (-N=N-) and phenyl rings. According to previous DFT results performed on system, *trans*-azobenzene–carbon dioxide showed [107] that CO_2_ molecules preferentially interact with the lone electron pair of the azo group (17.0 kJ mol^−1^, 4 × -N=N- groups per one MTA), and also favourable interactions are with the *π*-electron system of aromatic rings with maximal stabilization energy of 14.4 kJ mol^−1^ (12 × phenyl rings per one MTA linker).

**3. Pore volume and size effect**—3673.6 Å^3^ (47.8%) for **UPJS-15 (AC)** and 3103.5 Å^3^ (39.8%) for **UPJS-16 (AC)** of accessible volume according to SC-XRD (see Section 3.1), which are sufficient to provide attractive ion–dipole, dipole–dipole and van der Waals interactions.

**Figure 10:**
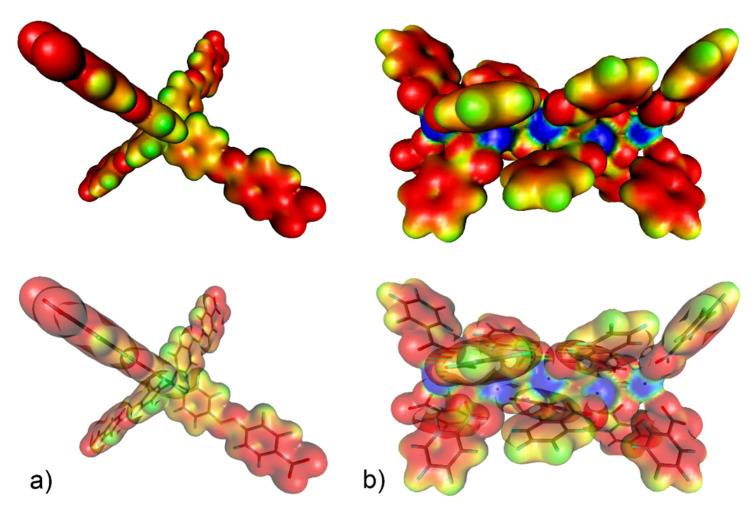
(**a**) Electron density isosurface (with 1% probability) colour-coded with the electrostatic potential (up) and location of the selected motifs of **UPJS-15** therein (down) for (**a**) MTA linker (the electrostatic colour values are: red < −0.2, yellow = −0.15, green = −0.1, pale blue = −0.05, and blue = 0.00) and (**b**) strontium(II) cluster (red < −0.10, yellow = −0.05, green = 0.00, pale blue = 0.05, and blue > 0.10).

**Figure 11:**
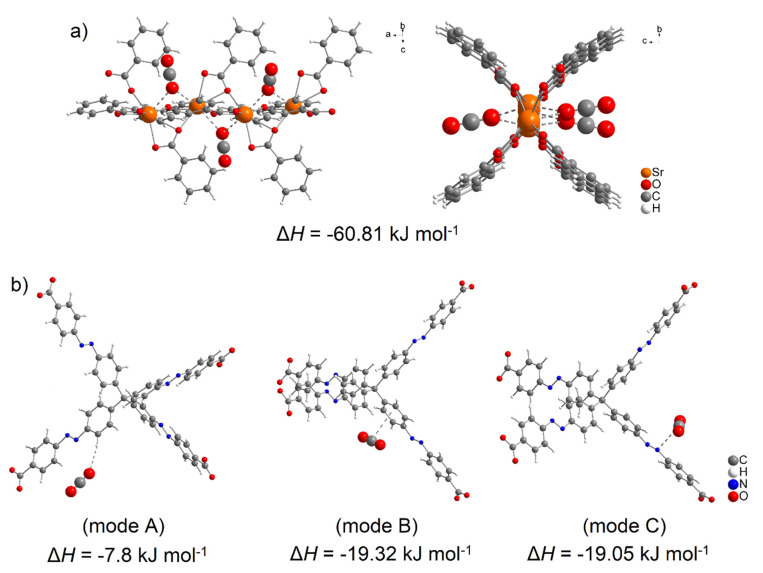
Molecular modelling of CO_2_ interaction with (**a**) strontium (II) cluster (representing CUSs) and (**b**) MTA linker using DFT calculation with corresponding adsorption enthalpies (mode A represents CO_2_ ··· π interaction, mode B is CO_2_ ··· H-C interaction within phenyl ring and mode C represents CO_2_ ··· -N=N- interaction of azo group).

DFT calculation is a powerful method used to explain the increased affinity of gas molecules to the porous frameworks in MOF materials, similar to the previous study focused on the adsorption of small gas molecules in Fe-based MOFs [108]. The increased CO_2_ storage capacity of **UPJS-15 (AC)** led us to investigate the active sites and affinities of carbon dioxide using DFT modelling, the results of which are depicted in Figure 10 and Figure 11. The proposed active sites mentioned above were studied first. As can be seen from Figure 10a, phenyl rings and azo bonds are potential sites of possible interaction with CO_2_ molecules within the MTA linker, as they show considerable electronegativity. Therefore, they are able to interact with a carbon atom in CO_2_ that has a partially positive charge (*δ*^+^). Based on previous studies, it is known that CUSs also serve as active sites for the adsorption of various molecules [32]. Therefore, the electron density isosurface of the Sr(II) cluster was studied, which in addition to possible interactions with phenyl rings, revealed electropositive sites in the cluster that are represented by free orbitals created by the elimination of coordinated water molecules in the material activation process. CUSs are able to interact with carbon dioxide through oxygen atoms that have a partially negative charge (*δ*^−^) within the CO_2_ molecule.

Using DFT calculations, the degree of CO_2_ interactions with the detected active sites were determined using counterpoint calculations, and obtained results are shown in Figure 11. CO_2_ with CUSs within the Sr(II) cluster shows the highest adsorption enthalpy with a value of Δ*H* = −60.81 kJ mol^−1^ for each carbon dioxide molecule. The calculated positions of CO_2_ molecules in the cluster in different views are shown in Figure 11a. Within the MTA linker (see Figure 11b), several possible active sites were detected, and the calculated enthalpy values can be arranged as follows: the lowest value of Δ*H* = −7.80 kJ mol^−1^ (mode A) was identified when the oxygen atom (*δ^-^*) of CO_2_ interacted with the hydrogen atom of the CH group (*δ^−^*), followed by the interactions of the phenyl ring (*δ^−^*, mode B) and the azo bond (*δ*^−^, mode C) with a carbon atom of CO_2_ (*δ^−^*), whose values are approximately the same, Δ*H* = 19.32 kJ mol^−1^ and Δ*H* = 19.05 kJ mol^−1^, respectively.

As experimental results, confirmed by DFT calculations, evidence interesting results regarding CO_2_ adsorption, we decided to perform ideal adsorbed solution theory (IAST) calculations and comparisons with other porous materials. The selectivity of CO_2_ over N_2_ was calculated by IAST in the pressure range of 5–100 kPa. The adsorption selectivities for 50:50 and 10:90 CO_2_/N_2_ mixtures were calculated using the pure component isotherm fits, described by:

SCO2/N2=xCO2xN2yCO2yN2

where $S_{CO2/N2}$ is the selectivity of CO_2_ in a binary mixture with N_2_, *x*, and *y* denoting the equilibrium mole fraction for CO_2_ in the adsorbed phase and gas phase, respectively [109,110]. The prediction of adsorption selectivities ($S_{CO2/N2}$) from the experimental carbon dioxide and nitrogen adsorption isotherms were fitted with a triple-site Langmuir equation [111,112]. Calculated selectivities of CO_2_ for equimolar and 10:90 CO_2_/N_2_ mixtures @ 0°C on **UPJS-15 (AC)** and **UPJS-16 (AC)** are depicted in Figure 9c. Because **UPJS-15 (AC)** adsorbed large amounts of nitrogen and carbon dioxide, the compound did not show significant selectivity, and the function is almost linear over the entire pressure range. The maximal value $S_{CO2/N2}\;@$ 5 kPa is 0.17 for the CO_2_/N_2_ 50:50 binary mixture and 0.02 for the CO_2_/N_2_ 10:90 mixture. For **UPJS-16 (AC)**, significant selectivity at low pressure was observed at 455 for the CO_2_/N_2_ equimolar binary mixture and 50 for the CO_2_/N_2_ 10:90 mixture, which decreases with increasing pressure to 20 and 3 @ 100 kPa, respectively. These results indicate that the material is able to selectively adsorb carbon dioxide at low pressure and low concentration, which is an important prerequisite for its practical use as a selective adsorbent of this greenhouse gas from the atmosphere for environmental application. The selectivity of **UPJS-16 (AC)** under CO_2_/N_2_ 50:50 condition exceeds known MOF materials, such as MOF-5, HKUST-1, ZIF-8, or MOF-74 [112,113]. To the best of our knowledge, it should be noted that selectivity is one of the highest within MOF materials. For this reason, further comparisons were performed with other porous materials. The calculated value is comparable to hierarchical porous carbon LCN2 (490) [114] or polyoxometalate-based ionic framework IF-Ni-Me (410) [115] and two times lower than N-doped carbon HAC-850 (850 @ 25 °C) [116]. The selectivity under CO_2_/N_2_ 10:90 conditions is comparable to materials BIT-72 [117], NKU-521 [118], and CAU-1 [117], whose values were calculated @ 0 °C using simulated post-combustion flue gas (CO_2_/N_2_ 15:85), are 49, 48, and 38, respectively. However, the calculated value is lower than the ones found for other porous materials, such as bio-MOF-13 (295), bio-MOF-12 (170), bio-MOF-11 (105) [118,119], polybenzoxazine surface-modified porous carbon (85–190) [120], and COF material N_4_CMP-4 (95) [121] under the same calculated conditions (CO_2_/N_2_ 10:90 @ 0 °C).

Hydrogen adsorption measurements revealed that compounds adsorbed only limited amounts of hydrogen (see Figure 9d), 21.1 cm^3^ g^−1^ (0.19 wt.%; 0.94 mmol g^−1^) for **UPJS-15 (AC)** and 4.3 cm^3^ g^−1^ (0.04 wt.%; 0.19 mmol g^−1^) for **UPJS-16 (AC)** @ −196 °C and 1 bar. It can be stated that among the mentioned MOF materials based on tetrahedral ligands, these are the lowest achieved values (see Table 2). Although the presented materials do not show an excellent hydrogen storage capacity, their high affinity for CO_2_ over H_2_ predetermines them for removal of carbon dioxide in reactions associated with hydrogen production, such as water–gas shift (WGS) and direct steam reforming (DSR) reactions [122]. Similar behaviour was also observed for compounds of the MOF-76 family [123].

### 3.4. SEM and EDS Analyses

**Figure 12:**
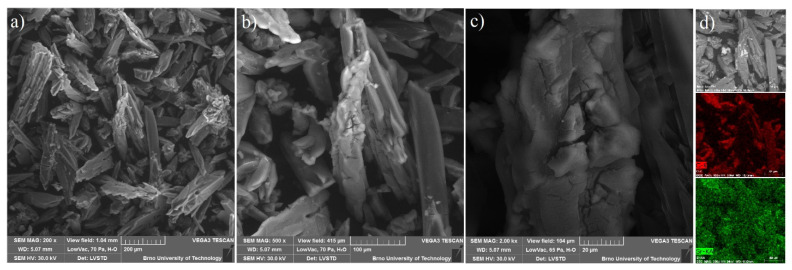
SEM figures of **UPJS-15 (AC)** surface mapping at the various fields of view: (**a**) 1.04 mm, (**b**) 415 µm, and (**c**) 104 µm. (**d**) EDS mapping of C (red colour) and Sr (green colour) distribution on crystals of **UPJS-15 (AC)**.

Based on the textural results of UPJS MOFs mentioned above, **UPJS-15** was selected as a host for sulphur in Li-S batteries due to its high porosity. **UPJS-15 (AC)** was investigated by energy-dispersive X-ray spectroscopy analysis (EDS) and scanning electron microscopy (SEM) to characterize the surface of the prepared material. SEM images of **UPJS-15 (AC)** at view fields of 1.04 mm, 415 µm, and 104 *µ*m are illustrated in Figure 12a–c. As can be seen from the described figures, the crystals of **UPJS-15 (AC)** are needle-shaped with a length of about 200 µm. EDS analysis (see Figure 12d) confirmed the presence of carbon and strontium on the surface of **UPJS-15 (AC)** crystals, and the distribution of both elements is uniform on the crystal’s surface.

**Figure 13:**
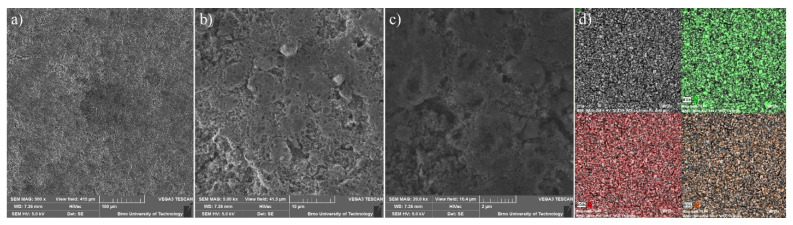
SEM at view fields of (**a**) 415 µm, (**b**) 41.5 µm, and (**c**) 10.4 µm, and (**d**) EDS mapping of S (green colour), C (red colour) and Sr (orange colour) of the **S/UPJS-15** cathode after pressing and before cycling experiments.

**Figure 14:**
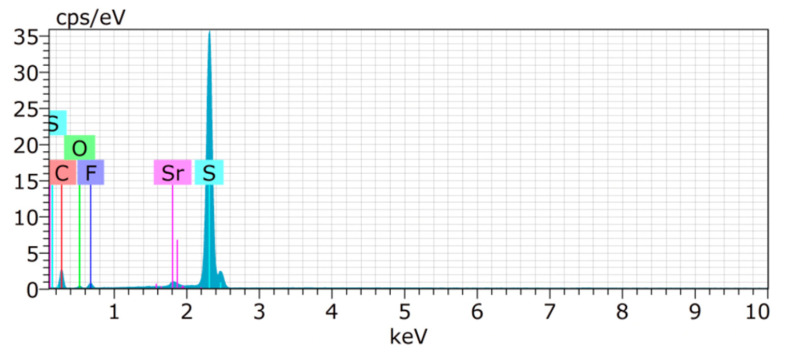
EDS spectrum of the **S/UPJS-15** electrode, containing detectable C (red), S (teal), F (violet), O (green), and Sr (pink) atoms.

For the constructed sulphur cathode, the structural properties on the particle’s surface are considered to be critical for its electrochemical performance. Therefore, the surface of **S/UPJS-15** composite was analyzed by SEM and EDS. The SEM images of the **S/UPJS-15** cathode after pressing are depicted in Figure 13a–c at view fields of 415 µm, 41.5 µm, and 10.4 µm. The electrode surface is uniform only with limited carks or holes and sufficiently porous for electrolyte penetration. The surface mapping of the **S/UPJS-15** electrode is shown in Figure 13d, and it can be seen that elements sulphur, carbon, and strontium are present and homogenously distributed on the electrode surface and the whole bulk of the material. The EDS spectra and corresponding exact element contents (C, F, S, O, and Sr) in the **S/UPJS-15** electrode are summarized in Table 3 and displayed in Figure 14. The carbon content (56.05 wt.%; 73.75 at.%) could be higher due to the application of the sample on the carbon support before measurement. The obtained content of sulphur (31.39 wt.%; 15.47 at.%) analyzed by EDS analysis indicates successful S incorporation into **UPJS-15** material. Fluorine (5.98 wt.%; 4.97 at.%) is present in the binder, PVDF, and strontium (0.87 wt.%; 0.16 at.%) represents a metal ion in the **UPJS-15** framework.

### 3.5. Electrochemical Properties

**Figure 15:**
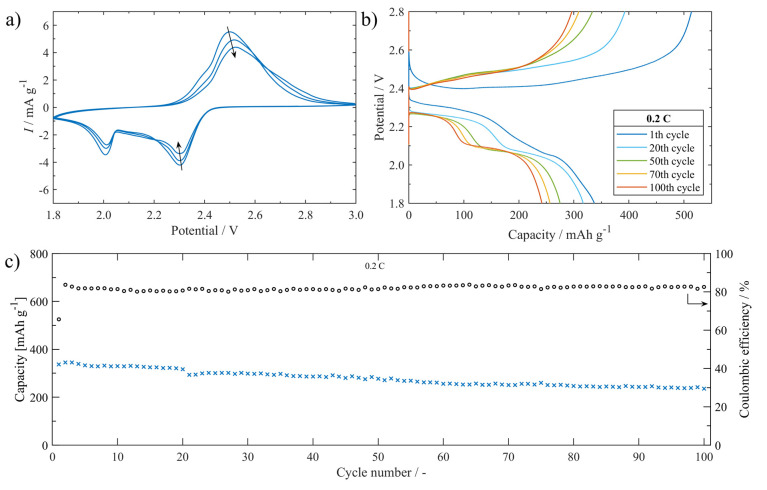
(**a**) The **S/UPJS-15** cathode cycling voltammogram between 1.8 and 3.0 V at 0.1 mV s−1. (**b**) Charge/discharge curves platform between 1.8 and 2.8 V during cycling at 0.2 C. (**c**) The cell cycling performance containing the **S/UPJS-15** cathode for 100 cycles at 0.2 C.

The electrochemical performance of the Li-S battery assembled with **S/UPJS-15** cathode was systematically investigated in electrochemical test cells. CV curves of the cells at 0.1 mV s^−1^ within the potential window 1.8–3.0 V are shown in Figure 15a. The typical redox reaction of Li-S batteries was accompanied by two prominent cathodic peaks located at around 2.3 V and 2.0 V. The first cathodic peak could be described as the phase transitions of sulphur to dissolved polysulphides (Li_2_S*_x_*, 4 ≤ *x* ≤ 8) and then to insoluble discharge products Li_2_S_2_/Li_2_S, respectively. The anodic scan is related to the reverse transformation reaction from short-chain polysulphides to long-chain polysulphides, and finally to sulphur. The intensity of CV curves is slightly decreasing with the number of cycles. The charge/discharge curves between the potential range of 1.8 and 2.8 V of the **S/UPJS-15** cathode at 0.2 C are presented in Figure 15b. The low-voltage plateau in the first cycle is depressed, though in other cycles both plateaus are visible with correspondence to the CV curves. The cycling performance at a cycling rate of 0.2 C during 100 cycles is depicted in Figure 15c. The initial discharge capacity was 337 mAh g^−1^ at 0.2 C, and decreased continuously to 235 mAh g^−1^ after 100 charge/discharge cycles. The capacity retention after 100 cycles was 69.8%, corresponding to a fading rate of 0.3% per cycle. Coulombic efficiency during the cycling procedure at 0.2 C reached a value of around 81.8%. Obtained initial discharge capacity is comparable to Mg-1,4-BDC [124], however, gently lower than HKUST-1 [125], RT-MOF-5 [126], Ce-UiO-66-BPDC [127], or MOF-5 [128]. It could be noted that although these materials show higher initial discharge capacities, the final capacity is significantly decreased during cycling and thus offers lower capacity retentions compared to **UPJS-15** (see Table 4).

In summary, it could be concluded that **UPJS-15** can be applied in Li-S batteries. By adjusting the electrode material preparation process, it will be possible to improve properties and achieve higher discharge capacities of the battery cell.

## 4. Conclusions

In this study, two new alkaline earth metal–organic frameworks (AE-MOFs) containing Sr(II) (**UPJS-15**)/Ba(II) (**UPJS-16**) ions and prolonged tetrahedral tetraazo-tetracarboxylic acid (H_4_MTA) were synthesized and investigated in detail in various fields of application. Single-crystal X-ray (SC-XRD) analysis revealed that the materials are isostructural and their crystal structures are constructed by alkaline–earth metal ions sequenced in 1D polymeric chains connected by MTA linkers. In their porous frameworks, 1D channels with sizes of about 11.19 × 10.10 Å^2^ for **UPJS-15 (AS)** and 10.69 × 9.70 Å^2^ for **UPJS-16 (AS)** are located. The porous channels are filled with solvents represented by DMF and H_2_O molecules. Solvents situated in the frameworks were solvent-exchanged for a lower boiling solvent, especially ethanol, for simpler and more effective activation. Synthesized AE-MOFs were studied by the combination of various analytical techniques EDS, SEM, HE-PXRD, PXRD, TG, DRIFT, IR, ICP-MAS, and EA. The compounds’ activation processes were investigated by a combination of in situ heating HE-PXRD, TG, and in situ heating DRIFT, which confirmed the stability of the materials’ frameworks after the degassing process. The samples were further investigated as adsorbents of different gases (Ar, N_2_, CO_2_, and H_2_), and the obtained adsorption outcomes were compared with other metal–organic frameworks consisting of tetrahedral linkers. Argon (@ −186 °C) and nitrogen (@ −196 °C) adsorption/desorption measurements demonstrated that the **UPJS-15** compound exhibits a *S_BET_* area of 1321 m^2^ g^−1^ (Ar)/1250 m^2^ g^−1^ (N_2_) and **UPJS-16** material adsorbs only a limited quantity of mentioned adsorbates. It should be noted that both materials are able to store CO_2_ molecules with maximum capacities of 22.4 wt.% (5.08 mmol g^−1^) @ 0 °C; 14.7 wt.% (3.34 mmol g^−1^) @ 20 °C and 101 kPa for **UPJS-15** and 11.5 wt.% (2.63 mmol g^−1^) @ 0 °C; 8.4 wt.% (1.89 mmol g^−1^) @ 20 °C and 101 kPa for **UPJS-16**. Moreover, DFT modelling was used to investigate the active adsorption sites within the alkaline earth central atoms and the MTA molecule (azo-functional group, C-H and π electron system of aromatic rings). The DFT results reveal that the primary adsorption sites are three coordinatively unsaturated sites (CUSs) located on the central atoms (Δ*H* = −60.81 kJ mol^−1^) and azo groups (Δ*H* = −19.05 kJ mol^−1^ for -N=N- ··· CO_2_ interaction)/phenyl-rings (Δ*H* = −19.32 kJ mol^−1^ for π ··· CO_2_ interaction and −Δ*H* = −7.80 kJ mol^−1^ for C-H ··· CO_2_ interaction) within the MTA linker. Based on the IAST calculation, **UPJS-16** showed considerable selectivity at low pressure with a maximum at 50 for CO_2_/N_2_ 10:90 mixture and 455 for CO_2_/N_2_ 50:50 mixture. Calculated results prove that **UPJS-16** could store and catch huge quantities of carbon dioxide from the air, even at low pressures. The mentioned measurements, calculations, and findings are especially important in the selective capture of CO_2_ and the reduction of its concentration in the air, which is crucial from the point of view of environmental applications for reducing global warming. From the point of view of energy storage, the materials were also studied as hydrogen adsorbents, but the measurements show only limited adsorbed quantities of H_2_ by prepared compounds: 0.19 wt.% (0.94 mmol g^−1^) for **UPJS-15** and 0.04 wt.% (0.19 mmol g^−1^) for **UPJS-16** at @ −196 °C and 1 bar. Although the materials adsorbed small amounts of hydrogen, it is also possible to find a positive in this result, which lies in the high selectivity of the CO_2_ and H_2_ separation. Because the purification of hydrogen in the mentioned gas mixture is important in many technological processes of hydrogen production, such as WGS and DSR reactions. The second way of using synthesized compounds in the field of energy storage presented in this study was the application of **UPJS-15** as a support for sulphur in a Li-S battery. The battery constructed from **UPJS-15** material showed an initial discharge capacity of 337 mAh g^−1^ at a cycling rate of 0.2 C, which decreased continuously slowly to 235 mAh g^−1^ after 100 charge/discharge cycles with a final capacity retention of ~70%. Although the material displayed a slightly lower final discharge capacity than other metal–organic framework materials, it has a higher capacity retention at the end of the cycling processes.

## Figures and Tables

**Table 1:** Assignment of principal IR bands in cm^−1^ to characteristic vibration in compounds **UPJS-15**, **UPJS-16**, and solvents.

Absorption Band	Sample								
	UPJS-15 (AS)	UPJS-16 (AS)	UPJS-15 (EX)	UPJS-16 (EX)	UPJS-15 (AC)	UPJS-16 (AC)	H_2_O	DMF	EtOH
*ν*(OH)	3380 (w)	3386 (w)	3331 (w)	3312 (w)	-	-	3320 (s)	-	3319 (m)
*ν*(CH)_ar_	3060 (w)	3053 (w)	3061 (w)	3059 (w)	3086 (w) 3064 (w) 3031 (w)	3086 (w) 3062 (w) 3030 (w)	-	-	-
*ν*(CH)_aliph_	2926 (w) 2853 (w)	2926 (w) 2850 (w)	2966 (w) 2923 (w) 2884 (w)	2965 (w) 2921 (w) 2887 (w)	-	-	-	2924 (w) 2856 (w)	2971 (m) 2921 (w) 2876 (w)
*ν*(C=O)	1649 (s)	1640 (s)	-	-	-	-	-	1656 (s)	-
*ν*(COO^−^)_as_	1591 (s)	1589 (m)	1591 (m)	1535 (s)	1589 (m)	1589 (m)	-	-	-
*ν*(C=C)_ar_	1543 (s) 1490 (m)	1544 (m) 1493 (w)	1535 (m) 1493 (w)	1493 (w)	1541 (m) 1491 (w)	1535 (m) 1494 (w)	-	-	-
*ν*(COO^−^)_s_	1385 (s)	1375 (s)	1384 (s)	1372 (s)	1386 (s)	1374 (s)	-	-	-
*δ*(COO^−^)	791 (s)	790 (s)	788 (s)	788 (s)	791 (s)	787 (s)	-	-	-

(s), strong; (m), medium; (w), weak; ar, aromatic; aliph, aliphatic; as, antisymmetric; and s, symmetric.

**Table 2:** *S_BET_* areas, CO_2_, and H_2_ storage capacities of MOFs containing linkers with tetraphenylmethane moiety.

Linker	Composition	Acronym	*N_2_ @ −196 °C*	*Ar @ −186 °C*	*H_2_ @ −196 °C*	*CO_2_*	*T*	*Ref.*
			*S_BET_* [m^2^ g^−1^]	*S_BET_* [m^2^ g^−1^]	*1 atm* *wt.%*	*1 atm* *wt.%*	[°C]	
*L1*	{[Sr_3_(*L1*)_3/2_]∙4DMF∙7H_2_O}_n_	UPJS-8	-	-	2.17	2.64	20	[39]
*L1*	{[Ca_4_(O)(*L1*)_3/2_(H_2_O)_4_]∙4DMF∙4H_2_O}_n_	UPJS-7	103	126	3.65	3.92	20	[39]
*L1*	{[Ni_2_(*L1*)(*L8*)_2_]·4DMF·8H_2_O}_n_	-	141	-	0.7	-	-	[88]
*L1*	{[Zn_2_(*L1*)(H_2_O)_2_]·3DMF·3H_2_O}_n_	UPJS-2	248	-	-	5.67 4.74	0 20	[89]
*L1*	{[Ni_2_(*L1*)(*L9*)_2_]·8H_2_O}_n_	-	288	-	-	4.06 2.29	0 20	[90]
*L1*	{[Ba_3_(*L1*)_3/2_(H_2_O)_6_]·2DMF·4H_2_O}_n_	UPJS-9	320	358	1.80	2.41	20	[39]
*L1*	{[Ni_2_(*L1*)(*L10*)_2_]·16H_2_O}_n_	-	355	-	-	3.53 2.25	0 20	[90]
*L1*	{[Co_4_(*L1*)_2_(H_2_O)_8_] ·13DMF·11H_2_O}_n_	SNU-15	356	-	0.74	7.02	0	[91]
*L1*	{[Cu_2_(*L1*)(H_2_O)_2_]·6DEF·2H_2_O}_n_	-	526	-	0.78	-	-	[92]
*L1*	{[Zn_2_(*L1*)(*L8*)_2_]·2DMF·7H_2_O}_n_	UPJS-4	-	644	1.28	10.5 7.0	0 20	[93]
*L1*	{[Ni_4_(*L1*)_2_(H_2_O)_8_]·10DMF·11H_2_O}_n_	UPJS-3	700	-	-	12.4 6.5	0 20	[94]
*L1*	{[Pb_4_(*L1*)_2_(H_2_O)_4_]·5DMF·H_2_O}_n_	UPJS-5	980	-	-	9.3	0	[95,96]
*L1*	{[Zr_6_(O)(OH)(*L1*)_2_(*L11*)_4_(H_2_O)_4_] ·xDMF·yMeOH}_n_	MOF-812	1390	-	-	-	-	[97]
*L2*	{[Cu_2_(*L2*)(DMF)_2_]·2DMF·4H_2_O}_n_	-	555	-	1.0	-	-	[98]
*L3*	{[Cu_2_(*L3*)(H_2_O)_2_]·14DMF·5H_2_O}_n_	-	791	-	1.26	-	-	[93]
*L3*	{[Co_5_(*L3*)(H*L3*)_2_]·15DMF·37H_2_O}_n_	-	965	-	1.44	14.9 9.3	0 20	[99]
*L3*	{[Zn_2_(H_2_O)_2_(*L3*)]·xG}_n_	-	1170	-	1.75	-	-	[100]
*L3*	{[Zn_4_(*L3*)(H_2_*L3*)]·12DEF·40H_2_O}_n_	-	1284	-	2.07	20.0 12.4	0 20	[99]
*L3*	{[Hf_6_(OH)_16_(*L3*)]·xG}_n_	PCN-523	1743	-	-	-	-	[101]
*L3*	{[Zr_6_(OH)_16_(*L3*)]·xG}_n_	PCN-521	3411	-	-	-	-	[101]
*L4*	{[Ba_2_(MTA)(H_2_O)]·2DMF·3H_2_O}_n_	UPJS-16	13	36	0.04	11.5 8.4	0 20	TA
*L4*	{[Cd_2_(MTA)]·5H_2_O·4DMF}_n_	UPJS-14	830	-	-	3.52	30	[70]
*L4*	{[Zn_2_(MTA)]·4H_2_O·3DMF}_n_	UPJS-13	1057	-	-	4.14	30	[70]
*L4*	{[Sr_2_(MTA)(H_2_O)]·H_2_O·4DMF}_n_	UPJS-15	1250	1321	0.19	22.4 14.7	0 20	TA
*L5*	{[Cu_2_(*L5*)(H_2_O)_2_]·12DEF·26H_2_O}_n_	-	262	-	1.1	-	-	[98]
*L6*	{[Cu_4_(*L6*)(H_2_O)_4_]·10DMF·OX·8H_2_O}_n_	NOTT-140	2620	-	2.5	14.1	20	[102]
*L7*	{[Cu_2_(H_4_*L7*)(H_2_O)_2_]·18DMF·5H_2_O}_n_	-	313	-	0.6	-	-	[103]
*L7*	{[Zn_12_(OH)_4_(H_2_O)_4_(*L7*)(H_2_*L7*)_2_] ·45DMF·44H_2_O}_n_	-	2718	-	2.8	-	-	[103]

*L1* = methanetetrabenzoic acid, *L2* = methanetetracinnamic acid, *L3* = 4,4′,4″,4‴-(4,4′,4″,4‴-methanetetrayltetrakis(benzene-4,1-diyl)tetrakis(ethyne-2,1-diyl))tetrabenzoic acid, *L4* = 4,4′,4″,4‴-(4,4′,4″,4‴-methanetetrayltetrakis(benzene-4,1-diyl)tetrakis(aza))tetrakis(methan-1-yl-1-yliden)tetrabenzoic acid, *L5* = 4,4′,4″,4‴-(4,4′,4″,4‴-methanetetrayltetrakis(benzene-4,1-diyl)tetrakis(ethine))tetrakis(methan-1-yl-1-yliden)tetrabenzoic acid, *L6* = 4,4′,4″,4‴-(4,4′,4″,4‴-methanetetrayltetrakis(benzene-4,1-diyl)tetrakis(ethyne-2,1-diyl))isophthalic acid, *L7* = f tetrakis{3,5-bis[(4-carboxyl)phenyl]phenyl}methane, *L8* = 1,4,8,11-tetraazacyclotetradecane, *L9* = 1,3,6,9,11,14-hexaazatricyclodecane, *L10* = hexaazatricycloeicosane, *L11* = formate, DMF = *N*,*N’*-dimethylformamide, DEF = *N*,*N*′-diethylformamide, MeOH = methanol, G = guest, OX = 1,4-dioxane, and TA = this article.

**Table 3:** The elemental composition of the **S/UPJS-15** electrode.

Element	Norm. Content [wt.%]	Atom. Content [at.%]
Carbon	56.05	73.75
Sulphur	31.39	15.47
Fluorine	5.98	4.97
Oxygen	5.72	5.65
Strontium	0.87	0.16

**Table 4:** List of MOF-based electrodes in Li-S batteries and their electrochemical properties.

MOF	Initial Capacity [mAh g^−1^]	Number of Cycles	Final Capacity [mAh g^−1^]	C-Rate	S Content [wt. %]	Capacity Retention [%]	Reference
RT-MOF-5	~540	40	~70	0.4 C	48	13.0	[126]
MOF-5	874	50	272	0.5 C	58	31.1	[128]
Ce-UiO-66-BPDC	~700	50	~300	0.1 C	25	42.3	[127]
Mg-1,4-BDC	392	100	180	0.2 C	69	46.0	[124]
HKUST-1	431	300	286	0.5 C	30	66.4	[125]
UPJS-15	337	100	235	0.2 C	60	69.8	this article

RT—room temperature synthesized, BPDC—biphenyldicarboxylate, BDC—1,4-benzenedicarboxylate.

## Supplementary Materials

The following supporting information can be downloaded at: https://www.mdpi.com/article/10.3390/nano13020234/s1, Table S1 Crystal structure refinement parameters for **UPJS-15 (AS)** and **UPJS-16 (AS)**; Table S2 Selected bond lengths [Å] and angles [°] for **UPJS-15 (AS)**; Table S3 Selected bond lengths [Å] and angles [°] for **UPJS-16 (AS)**; Figure S1 Measured PXRD patterns of final decomposition products and comparison with calculated patterns of SrCO3 and BaCO3.

## References

[1] Ghanbari T., Abnisa F., Wan Daud W.M. (2020). A Review on Production of Metal-organic Frameworks (MOF) for CO_2_ Adsorption. Sci. Total Environ..

[2] Connolly B.M., Madden D.G., Wheatley A.E., Fairen-Jimenez D. (2020). Shaping the Future of Fuel: Monolithic Metal–organic Frameworks for High-Density Gas Storage. J. Am. Chem. Soc..

[3] Almáši M. (2022). Current Development in MOFs for Hydrogen Storage. Metal-Organic Framework-Based Nanomaterials for Energy Conversion and Storage.

[4] Zelenka T., Simanova K., Saini R., Zelenkova G., Nehra S.P., Sharma A., Almasi M. (2022). Carbon Dioxide and Hydrogen Adsorption Study on Surface-modified HKUST-1 with Diamine/triamine. Sci. Rep..

[5] Li H., Li L., Lin R.-B., Zhou W., Zhang Z., Xiang S., Chen B. (2019). Porous Metal-organic Frameworks for Gas Storage and Separation: Status and Challenges. EnergyChem.

[6] Király N., Zeleňák V., Lenártová N., Zeleňáková A., Čižmár E., Almáši M., Meynen V., Hovan A., Gyepes R. (2021). Novel Lanthanide(III) Porphyrin-Based Metal–Organic Frameworks: Structure, Gas Adsorption, and Magnetic Properties. ACS Omega.

[7] Goetjen T.A., Liu J., Wu Y., Sui J., Zhang X., Hupp J.T., Farha O.K. (2020). Metal–Organic Framework (MOF) Materials as Polymerization Catalysts: A Review and Recent Advances. Chem. Commun..

[8] Ali M., Pervaiz E., Noor T., Rabi O., Zahra R., Yang M. (2020). Recent Advancements in MOF-Based Catalysts for Applications in Electrochemical and Photoelectrochemical Water Splitting: A Review. Int. J. Energy Res..

[9] Wang Q., Astruc D. (2019). State of the Art and Prospects in Metal–Organic Framework (MOF)-Based and MOF-Derived Nanocatalysis. Chem. Rev..

[10] Almáši M., Zeleňák V., Opanasenko M., Císařová I. (2015). Ce(III) and Lu(III) Metal–Organic Frameworks with Lewis Acid Metal Sites: Preparation, Sorption Properties and Catalytic Activity in Knoevenagel Condensation. Catal. Today.

[11] Lawson H.D., Walton S.P., Chan C. (2021). Metal–Organic Frameworks for Drug Delivery: A Design Perspective. ACS Appl. Mater. Interfaces.

[12] Cao J., Li X., Tian H. (2020). Metal-Organic Framework (MOF)-Based Drug Delivery. Curr. Med. Chem..

[13] Almáši M., Zeleňák V., Palotai P., Beňová E., Zeleňáková A. (2018). Metal-Organic Framework MIL-101(Fe)-NH_2_ Functionalized with Different Long-Chain Polyamines as Drug Delivery System. Inorg. Chem. Commun..

[14] Wang L., Yin K., Deng Q., Huang Q., He J., Duan J. (2022). Wetting Ridge-Guided Directional Water Self-Transport. Adv. Sci..

[15] He Y., Wang L., Wu T., Wu Z., Chen Y., Yin K. (2022). Facile Fabrication of Hierarchical Textures for Substrate-independent and Durable Superhydrophobic Surfaces. Nanoscale.

[16] Yin K., Chu D., Dong X., Wang C., Duan J.A., He J. (2017). Femtosecond Laser Induced Robust Periodic Nanoripple Structured Mesh for Highly Efficient Oil–water Separation. Nanoscale.

[17] Garg A., Almáši M., Bednarčík J., Sharma R., Rao V.S., Panchal P., Jain A., Sharma A. (2022). Gd(III) Metal-organic Framework as an Effective Humidity Sensor and its Hydrogen Adsorption Properties. Chemosphere.

[18] Wang L. (2020). Metal-Organic Frameworks for QCM-Based Gas Sensors: A Review. Sens. Actuators A Phys..

[19] Wu F., Ye J., Cao Y., Wang Z., Miao T., Shi Q. (2020). Recent Advances in Fluorescence Sensors Based on DNA–MOF Hybrids. Luminescence.

[20] Orts-Arroyo M., Rabelo R., Carrasco-Berlanga A., Moliner N., Cano J., Julve M., Lloret F., De Munno G., Ruiz-García R., Mayans J. (2021). Field-Induced Slow Magnetic Relaxation and Magnetocaloric Effects in an Oxalato-Bridged Gadolinium(III)-Based 2D MOF. Dalton Trans..

[21] Kim S., Oh H. (2020). Research Trend of Metal-Organic Frameworks for Magnetic Refrigeration Materials Application. Korean J. Mater. Res..

[22] Zeleňák V., Almáši M., Zeleňáková A., Hrubovčák P., Tarasenko R., Bourelly S., Llewellyn P. (2019). Large and Tunable Magnetocaloric Effect in Gadolinium-Organic Framework: Tuning by Solvent Exchange. Sci. Rep..

[23] Zhao H., Sheng L., Wang L., Xu H., He X. (2020). The Opportunity of Metal Organic Frameworks and Covalent Organic Frameworks in Lithium (ION) Batteries and Fuel Cells. Energy Storage Mater..

[24] Zhou L., Danilov D.L., Eichel R.A., Notten P.H. (2020). Host Materials Anchoring Polysulfides in Li–S Batteries Reviewed. Adv. Energy Mater..

[25] Capková D., Almáši M., Kazda T., Čech O., Király N., Čudek P., Fedorková A.S., Hornebecq V. (2020). Metal-Organic Framework Mil-101(Fe)–NH_2_ as an Efficient Host for Sulphur Storage in Long-Cycle Li–S Batteries. Electrochim. Acta.

[26] Capková D., Kazda T., Čech O., Király N., Zelenka T., Čudek P., Sharma A., Hornebecq V., Fedorková A.S., Almáši M. (2022). Influence of Metal-organic Framework MOF-76(Gd) Activation/carbonization on the Cycle Performance Stability in Li-S battery. J. Energy Storage.

[27] Qiao Z., Wang N., Jiang J., Zhou J. (2016). Design of Amine-Functionalized Metal–Organic Frameworks for CO_2_ Separation: The More Amine, the Better?. Chem. Commun..

[28] Klewiah I., Berawala D.S., Alexander Walker H.C., Andersen P.Ø., Nadeau P.H. (2020). Review of Experimental Sorption Studies of CO_2_ and CH_4_ in Shales. J. Nat. Gas Sci. Eng..

[29] Lee K., Howe J.D., Lin L.-C., Smit B., Neaton J.B. (2015). Small-Molecule Adsorption in Open-Site Metal–Organic Frameworks: A Systematic Density Functional Theory Study for Rational Design. Chem. Mater..

[30] Rada Z.H., Abid H.R., Sun H., Shang J., Li J., He Y., Liu S., Wang S. (2018). Effects of -NO_2_ and -NH_2_ Functional Groups in Mixed-Linker Zr-Based MOFs on Gas Adsorption of CO_2_ and CH_4_. Prog. Nat. Sci. Mater. Int..

[31] Pachfule P., Chen Y., Jiang J., Banerjee R. (2011). Fluorinated Metal-Organic Frameworks: Advantageous for Higher H_2_ and CO_2_ Adsorption or Not?. Chem.—Eur. J..

[32] Kökçam-Demir Ü., Goldman A., Esrafili L., Gharib M., Morsali A., Weingart O., Janiak C. (2020). Coordinatively Unsaturated Metal Sites (Open Metal Sites) in Metal–Organic Frameworks: Design and Applications. Chem. Soc. Rev..

[33] Caskey S.R., Wong-Foy A.G., Matzger A.J. (2008). Dramatic Tuning of Carbon Dioxide Uptake via Metal Substitution in a Coordination Polymer with Cylindrical Pores. J. Am. Chem. Soc..

[34] Milner P.J., Siegelman R.L., Forse A.C., Gonzalez M.I., Runčevski T., Martell J.D., Reimer J.A., Long J.R. (2017). A Diaminopropane-Appended Metal–Organic Framework Enabling Efficient CO_2_ Capture from Coal Flue Gas via a Mixed Adsorption Mechanism. J. Am. Chem. Soc..

[35] Kim E.J., Siegelman R.L., Jiang H.Z., Forse A.C., Lee J.-H., Martell J.D., Milner P.J., Falkowski J.M., Neaton J.B., Reimer J.A. (2020). Cooperative Carbon Capture and Steam Regeneration with Tetraamine-Appended Metal–Organic Frameworks. Science.

[36] Ren J., Langmi H.W., North B.C., Mathe M. (2014). Review on Processing of Metal-Organic Framework (MOF) Materials towards System Integration for Hydrogen Storage. Int. J. Energy Res..

[37] Rivard E., Trudeau M., Zaghib K. (2019). Hydrogen Storage for Mobility: A Review. Materials.

[38] Langmi H.W., Ren J., North B., Mathe M., Bessarabov D. (2014). Hydrogen Storage in Metal-Organic Frameworks: A Review. Electrochim. Acta.

[39] Almáši M., Zeleňák V., Gyepes R., Zauška Ľ., Bourrelly S. (2020). A Series of Four Novel Alkaline Earth Metal–Organic Frameworks Constructed of Ca(II), Sr(II), Ba(II) Ions and Tetrahedral MTB Linker: Structural Diversity, Stability Study and Low/High-Pressure Gas Adsorption Properties. RSC Adv..

[40] Sumida K., Hill M.R., Horike S., Dailly A., Long J.R. (2009). Synthesis and Hydrogen Storage Properties of Be_12_(OH)_12_(1,3,5-Benzenetribenzoate)_4_. J. Am. Chem. Soc..

[41] Lin R.-B., Li L., Zhou H.-L., Wu H., He C., Li S., Krishna R., Li J., Zhou W., Chen B. (2018). Molecular Sieving of Ethylene from Ethane Using a Rigid Metal–Organic Framework. Nat. Mater..

[42] Foo M.L., Horike S., Inubushi Y., Kitagawa S. (2012). An Alkaline Earth I_3_O_0_ Porous Coordination Polymer: [Ba_2_TMA(NO_3_)(DMF)]. Angew. Chem. Int. Ed..

[43] Liu T., Hu H., Ding X., Yuan H., Jin C., Nai J., Liu Y., Wang Y., Wan Y., Tao X. (2020). 12 Years Roadmap of the Sulfur Cathode for Lithium Sulfur Batteries (2009–2020). Energy Storage Mater..

[44] Kazda T., Capková D., Jaššo K., Fedorková Straková A., Shembel E., Markevich A., Sedlaříková M. (2021). Carrageenan as an Ecological Alternative of Polyvinylidene Difluoride Binder for Li-S Batteries. Materials.

[45] Capkova D., Kazda T., Čudek P., Strakova Fedorkova A. (2020). Binder Influence on Electrochemical Properties of Li-S Batteries. ECS Trans..

[46] Capková D., Kazda T., Straková Fedorková A., Čudek P., Oriňaková R. (2019). Carbon Materials as the Matrices for Sulfur in Li-S Batteries. ECS Trans..

[47] Liu G., Feng K., Cui H., Li J., Liu Y., Wang M. (2020). MOF Derived in-Situ Carbon-Encapsulated Fe_3_O_4_@C to Mediate Polysulfides Redox for Ultrastable Lithium-Sulfur Batteries. Chem. Eng. J..

[48] Jiang H., Liu X.-C., Wu Y., Shu Y., Gong X., Ke F.-S., Deng H. (2018). Metal-Organic Frameworks for High Charge-Discharge Rates in Lithium-Sulfur Batteries. Angew. Chem. Int. Ed..

[49] Li M., Feng W., Su W., Song C., Cheng L. (2019). Mof-Derived Hollow Cage Ni–Co Mixed Oxide/CNTS Nanocomposites with Enhanced Electrochemical Performance for Lithium–Sulfur Batteries. Ionics.

[50] Zhang H., Xin S., Li J., Cui H., Liu Y., Yang Y., Wang M. (2021). Synergistic Regulation of Polysulfides Immobilization and Conversion by MOF-Derived COP-HNC Nanocages for High-Performance Lithium-Sulfur Batteries. Nano Energy.

[51] Kazda T., Čudek P., Vondrák J., Sedlaříková M., Tichý J., Slávik M., Fafilek G., Čech O. (2017). Lithium-Sulphur Batteries Based on Biological 3D Structures. J. Solid State Electrochem..

[52] Fan L., Wu H., Wu X., Wang M., Cheng J., Zhang N., Feng Y., Sun K. (2019). Fe-MOF Derived Jujube Pit like Fe_3_O_4_/C Composite as Sulfur Host for Lithium-Sulfur Battery. Electrochim. Acta.

[53] Wang D., Zheng G., Zhang W., Niu X., Yan J., Nie T., Ji Z., Gu Y., Yan X. (2021). A Highly Stable Cathode for Lithium-Sulfur Battery Built of Ni-Doped Carbon Framework Linked to CNT. J. Alloys Compd..

[54] Geng P., Du M., Guo X., Pang H., Tian Z., Braunstein P., Xu Q. (2022). Bimetallic Metal-Organic Framework with High-Adsorption Capacity toward Lithium Polysulfides for Lithium–Sulfur Batteries. Energy Environ. Mater..

[55] Ye Y., Gong L., Xiang S., Zhang Z., Chen B. (2020). Metal–Organic Frameworks as a Versatile Platform for Proton Conductors. Adv. Mater..

[56] Li A.-L., Gao Q., Xu J., Bu X.-H. (2017). Proton-Conductive Metal-Organic Frameworks: Recent Advances and Perspectives. Coord. Chem. Rev..

[57] Afrin U., Mian M.R., Otake K.-I., Drout R.J., Redfern L.R., Horike S., Islamoglu T., Farha O.K. (2021). Proton Conductivity via Trapped Water in Phosphonate-Based Metal–Organic Frameworks Synthesized in Aqueous Media. Inorg. Chem..

[58] Chand S., Pal S.C., Lim D.-W., Otsubo K., Pal A., Kitagawa H., Das M.C. (2020). A 2d Mg(II)-MOF with High Density of Coordinated Waters as Sole Intrinsic Proton Sources for Ultrahigh Superprotonic Conduction. ACS Mater. Lett..

[59] Chen W., Wang J., Zhao L., Dai W., Li Z., Li G. (2018). Enhancing Proton Conductivity of a Highly Water Stable 3D Sr(II) Metal-Organic Framework by Exposure to Aqua-Ammonia Vapor. J. Alloys Compd..

[60] Saha D., Sen R., Maity T., Koner S. (2012). Porous Magnesium Carboxylate Framework: Synthesis, X-Ray Crystal Structure, Gas Adsorption Property and Heterogeneous Catalytic Aldol Condensation Reaction. Dalton Trans..

[61] Saha D., Maity T., Das S., Koner S. (2013). A Magnesium-Based Multifunctional Metal–Organic Framework: Synthesis, Thermally Induced Structural Variation, Selective Gas Adsorption, Photoluminescence and Heterogeneous Catalytic Study. Dalton Trans..

[62] Saha D., Maity T., Koner S. (2014). Alkaline Earth Metal-Based Metal–Organic Framework: Hydrothermal Synthesis, X-Ray Structure and Heterogeneously Catalyzed Claisen–Schmidt Reaction. Dalton Trans..

[63] Platero Prats A.E., de la Peña-O’Shea V.A., Iglesias M., Snejko N., Monge Á., Gutiérrez-Puebla E. (2010). Heterogeneous Catalysis with Alkaline-Earth Metal-Based MOFs: A Green Calcium Catalyst. ChemCatChem.

[64] Lacroix P.G., Malfant I., Lepetit C. (2016). Second-Order Nonlinear Optics in Coordination Chemistry: An Open Door towards Multi-Functional Materials and Molecular Switches. Coord. Chem. Rev..

[65] Song Y., Feng M.-L., Wu Z.-F., Huang X.-Y. (2015). Solvent-Assisted Construction of Diverse Mg-TDC Coordination Polymers. CrystEngComm.

[66] Liu D., Kramer S.A., Huxford-Phillips R.C., Wang S., Della Rocca J., Lin W. (2012). Coercing Bisphosphonates to Kill Cancer Cells with Nanoscale Coordination Polymers. Chem. Commun..

[67] Matlinska M.A., Ha M., Hughton B., Oliynyk A.O., Iyer A.K., Bernard G.M., Lambkin G., Lawrence M.C., Katz M.J., Mar A. (2019). Alkaline Earth Metal–Organic Frameworks with Tailorable Ion Release: A Path for Supporting Biomineralization. ACS Appl. Mater. Interfaces.

[68] Li Z., Li Z., Li S., Wang K., Ma F., Tang B. (2020). Potential Application Development of Sr/HCOOH Metal Organic Framework in Osteoarthritis. Microporous Mesoporous Mater..

[69] Almáši M. (2021). A Review on State of Art and Perspectives of Metal-Organic Frameworks (MOFs) in the Fight against Coronavirus SARS-CoV-2. J. Coord. Chem..

[70] Almáši M., Király N., Zeleňák V., Vilková M., Bourrelly S. (2021). Zinc(II) and Cadmium(II) Amorphous Metal–Organic Frameworks (AMOFs): Study of Activation Process and High-Pressure Adsorption of Greenhouse Gases. RSC Adv..

[71] Hammersley A.P., Svensson S.O., Hanfland M., Fitch A.N., Hausermann D. (1996). Two-Dimensional Detector Software: From Real Detector to Idealised Image or Two-Theta Scan. High Press. Res..

[72] Sheldrick G.M. (2015). Crystal Structure Refinement with SHELXL. Acta Crystallogr. Sect. C Struct. Chem..

[73] Spek A.L. (2009). Structure Validation in Chemical Crystallography. Acta Crystallogr. Sect. D Biol. Crystallogr..

[74] Brandenburg K. (2010). DIAMOND 3.2e.

[75] Alexandrov E.V., Shevchenko A.P., Blatov V.A. (2019). Topological Databases: Why Do We Need Them for Design of Coordination Polymers?. Cryst. Growth Des..

[76] Shevchenko A.P., Blatov V.A. (2021). Simplify to Understand: How to Elucidate Crystal Structures?. Struct. Chem..

[77] Frisch M.J., Trucks G., Schlegel H., Scuseria G., Robb M., Cheeseman J., Scalmani G., Barone V., Petersson G., Nakatsuji H. (2019).

[78] Becke A.D. (1993). Density-Functional Thermochemistry. III. The Role of Exact Exchange. J. Chem. Phys..

[79] Lee C., Yang W., Parr R.G. (1988). Development of the Colle-Salvetti Correlation-Energy Formula into a Functional of the Electron Density. Phys. Rev. B.

[80] Vosko S.H., Wilk L., Nusair M. (1980). Accurate Spin-Dependent Electron Liquid Correlation Energies for Local Spin Density Calculations: A Critical Analysis. Can. J. Phys..

[81] Fuentealba P., Preuss H., Stoll H., Von Szentpály L. (1982). A Proper Account of Core-Polarization with Pseudopotentials: Single Valence-Electron Alkali Compounds. Chem. Phys. Lett..

[82] Grimme S., Antony J., Ehrlich S., Krieg H. (2010). A Consistent and Accurate Ab Initio Parametrization of Density Functional Dispersion Correction (DFT-D) for the 94 Elements H-Pu. J. Chem. Phys..

[83] Groom C.R., Bruno I.J., Lightfoot M.P., Ward S.C. (2016). The Cambridge Structural Database. Acta Crystallogr. Sect. B Struct. Sci. Cryst. Eng. Mater..

[84] The Materials Project (2020). Materials Data on SrCO3 by Materials Project. https://materialsproject.org/materials/mp-33746.

[85] Antao S.M., Hassan I. (2007). BaCO_3_: High-Temperature Crystal Structures and the P*_mcm_*→R*_3m_* Phase Transition at 811 °C. Phys. Chem. Miner..

[86] Kitagawa S., Uemura K. (2005). Dynamic Porous Properties of Coordination Polymers Inspired by Hydrogen Bonds. Chem. Soc. Rev..

[87] Thommes M., Kaneko K., Neimark A.V., Olivier J.P., Rodriguez-Reinoso F., Rouquerol J., Sing K.S.W. (2015). Physisorption of Gases, with Special Reference to the Evaluation of Surface Area and Pore Size Distribution (IUPAC Technical Report). Pure Appl. Chem..

[88] Cheon Y.E., Suh M.P. (2008). Multifunctional Fourfold Interpenetrating Diamondoid Network: Gas Separation and Fabrication of Palladium Nanoparticles. Chem.—Eur. J..

[89] Almáši M., Zeleňák V., Gyepes R., Zukal A., Čejka J. (2013). Synthesis, Characterization and Sorption Properties of Zinc(II) Metal–Organic Framework Containing Methanetetrabenzoate Ligand. Colloids Surf. A Physicochem. Eng. Asp..

[90] Jiang X., Kou H.-Z. (2016). Solid State Reconstructive Phase Transition from Porous Supramolecular Network to Porous Coordination Polymer. Chem. Commun..

[91] Cheon Y.E., Suh M.P. (2009). Selective Gas Adsorption in a Microporous Metal–Organic Framework Constructed of Co^II^_4_ Clusters. Chem. Commun..

[92] Ma L., Jin A., Xie Z., Lin W. (2009). Freeze Drying Significantly Increases Permanent Porosity and Hydrogen Uptake in 4,4-Connected Metal-Organic Frameworks. Angew. Chem. Int. Ed..

[93] Almáši M., Zeleňák V., Zukal A., Kuchár J., Čejka J. (2016). A Novel Zinc(II) Metal–Organic Framework with a Diamond-like Structure: Synthesis, Study of Thermal Robustness and Gas Adsorption Properties. Dalton Trans..

[94] Almáši M., Zeleňák V., Opanasenko M., Čejka J. (2014). A Novel Nickel Metal–Organic Framework with Fluorite-like Structure: Gas Adsorption Properties and Catalytic Activity in Knoevenagel Condensation. Dalton Trans..

[95] Almáši M., Zeleňák V., Gyepes R., Bourrelly S., Opanasenko M.V., Llewellyn P.L., Čejka J. (2018). Microporous Lead–Organic Framework for Selective CO_2_ Adsorption and Heterogeneous Catalysis. Inorg. Chem..

[96] Almáši M., Zeleňák V., Opanasenko M.V., Čejka J. (2018). Efficient and Reusable Pb(II) Metal–Organic Framework for Knoevenagel Condensation. Catal. Lett..

[97] Furukawa H., Gándara F., Zhang Y.-B., Jiang J., Queen W.L., Hudson M.R., Yaghi O.M. (2014). Water Adsorption in Porous Metal–Organic Frameworks and Related Materials. J. Am. Chem. Soc..

[98] Liu D., Xie Z., Ma L., Lin W. (2010). Three-Dimensional Metal−Organic Frameworks Based on Tetrahedral and Square-Planar Building Blocks: Hydrogen Sorption and Dye Uptake Studies. Inorg. Chem..

[99] Wen L., Cheng P., Lin W. (2012). Mixed-Motif Interpenetration and Cross-Linking of High-Connectivity Networks Led to Robust and Porous Metal–Organic Frameworks with High Gas Uptake Capacities. Chem. Sci..

[100] Wen L., Cheng P., Lin W. (2012). Solvent-Induced Single-Crystal to Single-Crystal Transformation of a 2D Coordination Network to a 3D Metal–Organic Framework Greatly Enhances Porosity and Hydrogen Uptake. Chem. Commun..

[101] Zhang M., Chen Y.-P., Bosch M., Gentle T., Wang K., Feng D., Wang Z.U., Zhou H.-C. (2013). Symmetry-Guided Synthesis of Highly Porous Metal-Organic Frameworks with Fluorite Topology. Angew. Chem. Int. Ed..

[102] Tan C., Yang S., Champness N.R., Lin X., Blake A.J., Lewis W., Schröder M. (2011). High Capacity Gas Storage by a 4,8-Connected Metal–Organic Polyhedral Framework. Chem. Commun..

[103] Liu D., Wu H., Wang S., Xie Z., Li J., Lin W. (2012). A High Connectivity Metal–Organic Framework with Exceptional Hydrogen and Methane Uptake Capacities. Chem. Sci..

[104] Si X., Jiao C., Li F., Zhang J., Wang S., Liu S., Li Z., Sun L., Xu F., Gabelica Z. (2011). High and Selective CO_2_ Uptake, H_2_ Storage and Methanol Sensing on the Amine-Decorated 12-Connected Mof Cau-1. Energy Environ. Sci..

[105] Lee Y.-G., Moon H.R., Cheon Y.E., Suh M.P. (2008). A Comparison of the H_2_ Sorption Capacities of Isostructural Metal-Organic Frameworks with and without Accessible Metal Sites: [{Zn_2_(ABTC)(DMF)_2_}_3_] and [{Cu_2_(ABTC)(DDMF)_2_}_3_] versus [{Cu_2_(ABTC)}_3_]. Angew. Chem. Int. Ed..

[106] Kim T.K., Suh M.P. (2011). Selective CO_2_ Adsorption in a Flexible Non-Interpenetrated Metal–Organic Framework. Chem. Commun..

[107] Patel H.A., Hyun Je S., Park J., Chen D.P., Jung Y., Yavuz C.T., Coskun A. (2013). Unprecedented High-Temperature CO_2_ Selectivity in N_2_-Phobic Nanoporous Covalent Organic Polymers. Nat. Commun..

[108] Poater J., Gimferrer M., Poater A. (2018). Covalent and Ionic Capacity of MOFs To Sorb Small Gas Molecules. Inorg. Chem..

[109] Myers A.L., Prausnitz J.M. (1965). Thermodynamics of Mixed-Gas Adsorption. AIChE J..

[110] Langmuir I. (1918). The Adsorption of Gases on Plane Surfaces of Glass, Mica and Platinum. J. Am. Chem. Soc..

[111] Hay P.J., Wadt W.R. (1985). Ab Initio Effective Core Potentials for Molecular Calculations. Potentials for K to Au Including the Outermost Core Orbitals. J. Chem. Phys..

[112] Simmons J.M., Wu H., Zhou W., Yildirim T. (2011). Carbon Capture in Metal–Organic Frameworks—A Comparative Study. Energy Environ. Sci..

[113] Sumida K., Rogow D.L., Mason J.A., McDonald T.M., Bloch E.D., Herm Z.R., Bae T.-H., Long J.R. (2011). Carbon Dioxide Capture in Metal–Organic Frameworks. Chem. Rev..

[114] Saha D., Van Bramer S.E., Orkoulas G., Ho H.-C., Chen J., Henley D.K. (2017). CO_2_ Capture in Lignin-Derived and Nitrogen-Doped Hierarchical Porous Carbons. Carbon.

[115] Duan F., Liu X., Qu D., Li B., Wu L. (2021). Polyoxometalate-Based Ionic Frameworks for Highly Selective CO_2_ Capture and Separation. CCS Chem..

[116] Yang Z., Zhang G., Guo X., Xu Y. (2020). Designing a Novel N-Doped Adsorbent with Ultrahigh Selectivity for CO_2_: Waste Biomass Pyrolysis and Two-Step Activation. Biomass Convers. Biorefin..

[117] Li H., Feng X., Ma D., Zhang M., Zhang Y., Liu Y., Zhang J., Wang B. (2018). Stable Aluminum Metal–Organic Frameworks (Al-MOFs) for Balanced CO_2_ and Water Selectivity. ACS Appl. Mater. Interfaces.

[118] Li N., Chang Z., Huang H., Feng R., He W.W., Zhong M., Madden D.G., Zaworotko M.J., Bu X.H. (2019). CO_2_ Capture: Specific K^+^ Binding Sites as CO_2_ Traps in a Porous MOF for Enhenced CO_2_ Selective Sorption. Small.

[119] Li T., Chen D.-L., Sullivan J.E., Kozlowski M.T., Johnson J.K., Rosi N.L. (2013). Systematic Modulation and Enhancement of CO_2_: N_2_ Selectivity and Water Stability in an Isoreticular Series of Bio-MOF-11 Analogues. Chem. Sci..

[120] Hong L., Ju S., Liu X., Zhuang Q., Zhan G., Yu X. (2019). Highly Selective CO_2_ Uptake in Novel Fishnet-like Polybenzoxazine-Based Porous Carbon. Energy Fuels.

[121] Li G., Qin L., Yao C., Xu Y. (2017). Controlled Synthesis of Conjugated Polycarbazole Polymers via Structure Tuning for Gas Storage and Separation Applications. Sci. Rep..

[122] Idriss H., Scott M., Subramani V. (2015). Introduction to Hydrogen and Its Properties. Compendium of Hydrogen Energy.

[123] Garg A., Almáši M., Rattan Paul D., Poonia E., Luthra J.R., Sharma A. (2021). Metal-Organic Framework MOF-76(Nd): Synthesis, Characterization, and Study of Hydrogen Storage and Humidity Sensing. Front. Energy Res..

[124] Dhawa T., Chattopadhyay S., De G., Mahanty S. (2017). In Situ Mg/MgO-Embedded Mesoporous Carbon Derived from Magnesium 1,4-Benzenedicarboxylate Metal Organic Framework as Sustainable Li–S Battery Cathode Support. ACS Omega.

[125] Zhou J., Li R., Fan X., Chen Y., Han R., Li W., Zheng J., Wang B., Li X. (2014). Rational Design of a Metal–Organic Framework Host for Sulfur Storage in Fast, Long-Cycle Li–S Batteries. Energy Environ. Sci..

[126] Xi K., Cao S., Peng X., Ducati C., Vasant Kumar R., Cheetham A.K. (2013). Carbon with Hierarchical Pores from Carbonized Metal–Organic Frameworks for Lithium Sulphur Batteries. Chem. Commun..

[127] Chen X., Zhang M., Zhu J., Wang J., Jiao Z., Li Y. (2022). Boosting Electrochemical Performance of Li-S Batteries by Cerium-Based MOFs Coated with Polypyrrole. J. Alloys Compd..

[128] Bao W., Zhang Z., Zhou C., Lai Y., Li J. (2014). Multi-Walled Carbon Nanotubes @ Mesoporous Carbon Hybrid Nanocomposites from Carbonized Mitli-Walled Carbon Nanotubes @ Metal-Organic Framework for Lithium Sulphur Battery. J. Power Sources.

