# Supplementary material for: Sr(II) and Ba(II) Alkaline Earth Metal–Organic Frameworks (AE-MOFs) for Selective Gas Adsorption, Energy Storage, and Environmental Application
Source: Nanomaterials (Basel). 2023 Jan 4;13(2):234. doi: 10.3390/nano13020234 (PMC9866501; doi:10.3390/nano13020234)
Supplement: Supplementary file 1 [file nanomaterials-13-00234-s001.zip › nanomaterials-2130478-supplementary.pdf]

Electronic Supporting Information

(ESI)

Sr(II) and Ba(II) Alkaline Earth Metal–Organic Frameworks (AE-MOFs) for Selective Gas Adsorption, Energy Storage, and Environmental Application

Nikolas Király [1], Dominika Capková [2], Róbert Gyepes [3], Nikola Vargová [1], Tomáš Kazda [4], Jozef Bednarčík [5,6], Daria Yudina [5], Tomáš Zelenka [7], Pavel Čudek [4], Vladimír Zeleňák [1], Anshu Sharma [8], Vera Meynen [9], Virginie Hornebecq [10], Andrea Straková Fedorková [2] and Miroslav Almáši [1,*]

[1] Department of Inorganic Chemistry, Faculty of Science, Pavol Jozef Šafárik University in Košice, Moyzesova 11, SK-041 54 Košice, Slovakia
[2] Department of Physical Chemistry, Faculty of Sciences, Pavol Jozef Šafárik University in Košice, Moyzesova 11, SK-041 54 Košice, Slovakia
[3] Department of Inorganic Chemistry, Faculty of Science, Charles University, Albertov 8, CZ-128 43 Prague, Czech Republic
[4] Department of Electrical and Electronic Technology, Faculty of Electrical Engineering and Communication, Brno University of Technology, Technická 10, CZ-616 00 Brno, Czech Republic
[5] Department of Physics, Faculty of Science, Pavol Jozef Šafárik University in Košice, Park Angelinum 9, SK-041 01 Košice, Slovakia
[6] Institute of Experimental Physics, Slovak Academy of Sciences, Watsonova 47, SK-040 01 Košice, Slovakia
[7] Department of Chemistry, Faculty of Science, University of Ostrava, 30. Dubna 22, CZ-702 00 Ostrava, Czech Republic
[8] Department of Physics, School of Engineering & Technology, Central University of Haryana, Mahendergarh 123031, India
[9] Laboratory of Adsorption and Catalysis, University of Antwerp, Universiteitsplein 1, B-2610 Wilrijk, Belgium
[10] Centre National de la Recherche Scientifique (CNRS), Matériaux Divisé, Interfaces, Réactivité, Electrochimie (MADIREL), Centre de Saint Jérôme, Aix-Marseille University, Avenue Escadrille-Normandie-Niemen, F-133 97 Marseille, France
* Correspondence: miroslav.almasi@upjs.sk

Tables:

Table S1 Crystal structure refinement parameters for **UPJS-15 (AS)** and **UPJS-16 (AS)**.

**Identification code**	**UPJS-15 (AS)**	**UPJS-16 (AS)**
**Empirical formula**	C53 H32 N8 O10 Sr2	C53 H32 N8 O10 Ba2
**Formula weight**	1116.10	1215.54
**Temperature**	120(2) K	122(2) K
**Wavelength**	1.54178 Å	1.54178 Å
**Crystal system**	Tetragonal	Tetragonal
**Space group**	*I*-42d	*I*-42d
**Unit cell dimensions**	*a* = 32.3789(8) Å	*a* = 31.6708(9) Å
	b = 32.3789(8) Å	*b* = 31.6708(9) Å
	c =7.3286(6) Å	*c* = 7.7689(2) Å
**Volume**	7683.3(4) $Å^3$	7792.5(5) $Å^3$
Z	4	4
**Density (calculated)**	0.965 g cm^{-3}	1.036 g cm^{-3}
**Absorption coefficient**	2.197 mm^{-1}	8.172 mm^{-1}
***F*(000)**	2248	2392
**Crystal size**	0.367 × 0.106 × 0.076 mm^3	0.219 × 0.103 × 0.074 mm^3
**Theta range for data collection**	2.729 to 72.279°.	3.948 to 74.716°
**Index ranges**	-39 ≤ h ≤ 39, -39 ≤ k ≤ 40, -9 ≤ l ≤8	-38 ≤ h ≤ 39, -39 ≤ k ≤ 39, -9 ≤ l ≤ 9
**Reflections collected**	85094	52008
**Completeness to theta = 67.679°**	99.9 %	99.8 %
**Refinement method**	Full-matrix least-squares on F^2	Full-matrix least-squares on F^2
**Data / restraints / parameters**	3800 / 0 / 167	3996 / 0 / 159
**Goodness-of-fit on F^2**	1.048	1.012
**Final *R* indices [*I*>2sigma(*I*)]**	R_1 = 0.0449, wR_2 = 0.1195	R_1 = 0.0637, wR_2 = 0.1488
***R* indices (all data)**	R_1 = 0.0543, wR_2 = 0.1272	R_1 = 0.1189, wR_2 = 0.1832
**Extinction coefficient**	n/a	n/a
**Largest diff. peak and hole**	1.171and -0.396 e $Å^{-3}$	0.659 and -0.685 e A^{-3}
**CCDC deposition number**	2117419	2117419

Table S2 Selected bond lengths [Å] and angles [°] for **UPJS-15 (AS)**.

**Bond lengths**		**Bond angles**		**Bond angles**	
Sr(1)-O(1)#1	2.499(4)	O(1)#1-Sr(1)-O(1)	79.3(2)	O(2)#3-Sr(1)-O(2)#2	89.0(4)

Sr(1)-O(1)	2.499(4)	O(1)#1-Sr(1)-O(1)#2	71.39(16)	O(1)#1-Sr(1)-O(3)#2	71.78(12)
Sr(1)-O(1)#2	2.590(4)	O(1)-Sr(1)-O(1)#2	148.77(11)	O(1)-Sr(1)-O(3)#2	90.68(10)
Sr(1)-O(1)#3	2.590(4)	O(1)#2-Sr(1)-O(1)#3	139.27(18)	O(1)#2-Sr(1)-O(3)#2	70.39(11)
Sr(1)-O(2)#3	2.596(5)	O(1)#1-Sr(1)-O(2)#3	151.66(18)	O(1)#3-Sr(1)-O(3)#2	118.13(13)
Sr(1)-O(2)#2	2.596(5)	O(1)-Sr(1)-O(2)#3	102.60(19)	O(2)#3-Sr(1)-O(3)	116.98(14)
Sr(1)-O(3)#2	2.621(4)	O(1)#2-Sr(1)-O(2)#3	98.34(16)	O(2)#2-Sr(1)-O(3)	79.91(17)
Sr(1)-O(3)	2.621(4)	O(1)#3-Sr(1)-O(2)#3	50.11(13)	O(3)#2-Sr(1)-O(3)	157.48(4)

Symmetry transformations used to generate equivalent atoms: #1 x+0,-y+1/2,-z+5/4; #2 -x+1/2,-y+1/2,z-1/2; #3 -x+1/2,y,-z+7/4; #4 -x+1/2,-y+1/2,z+1/2.

Table S3 Selected bond lengths [Å] and angles [°] for **UPJS-16 (AS)**.

**Bond lengths**		**Bond angles**		**Bond angles**	
Ba(1)-O(1)#1	2.676(9)	O(1)#1-Ba(1)-O(1)	74.6(4)	O(2B)#3-Ba(1)-O(3)	86.7(5)
Ba(1)-O(1)	2.677(9)	O(1)#1-Ba(1)-O(2B)#2	157.0(5)	O(1)#3-Ba(1)-O(3)	118.7(3)
Ba(1)-O(2B)#2	2.71(2)	O(1)-Ba(1)-O(2B)#2	101.3(5)	O(1)#2-Ba(1)-O(3)	69.8(3)
Ba(1)-O(2B)#3	2.71(2)	O(1)#1-Ba(1)-O(1)#3	69.7(4)	O(1)#1-Ba(1)-O(2A)#2	138.0(5)
Ba(1)-O(1)#3	2.737(8)	O(1)-Ba(1)-O(1)#3	142.1(2)	O(1)-Ba(1)-O(2A)#2	82.0(5)
Ba(1)-O(1)#2	2.737(8)	O(1)#3-Ba(1)-O(1)#2	147.8(5)	O(1)#3-Ba(1)-O(2A)#2	117.3(5)
Ba(1)-O(3)	2.842(11)	O(1)#1-Ba(1)-O(3)	87.1(2)	O(1)#2-Ba(1)-O(2A)#2	47.9(5)
Ba(1)-O(3)#3	2.842(11)	O(1)-Ba(1)-O(3)	70.6(3)	O(3)#3-Ba(1)-O(2A)#2	73.9(5)
		O(2B)#2-Ba(1)-O(3)	113.3(5)	O(2A)#2-Ba(1)-O(2A)#3	135.3(9)

Symmetry transformations used to generate equivalent atoms: #1 -x+3/2,y,-z+3/4; #2 x+0,-y+3/2,-z+1/4 ; #3 -x+3/2,-y+3/2,z+1/2.

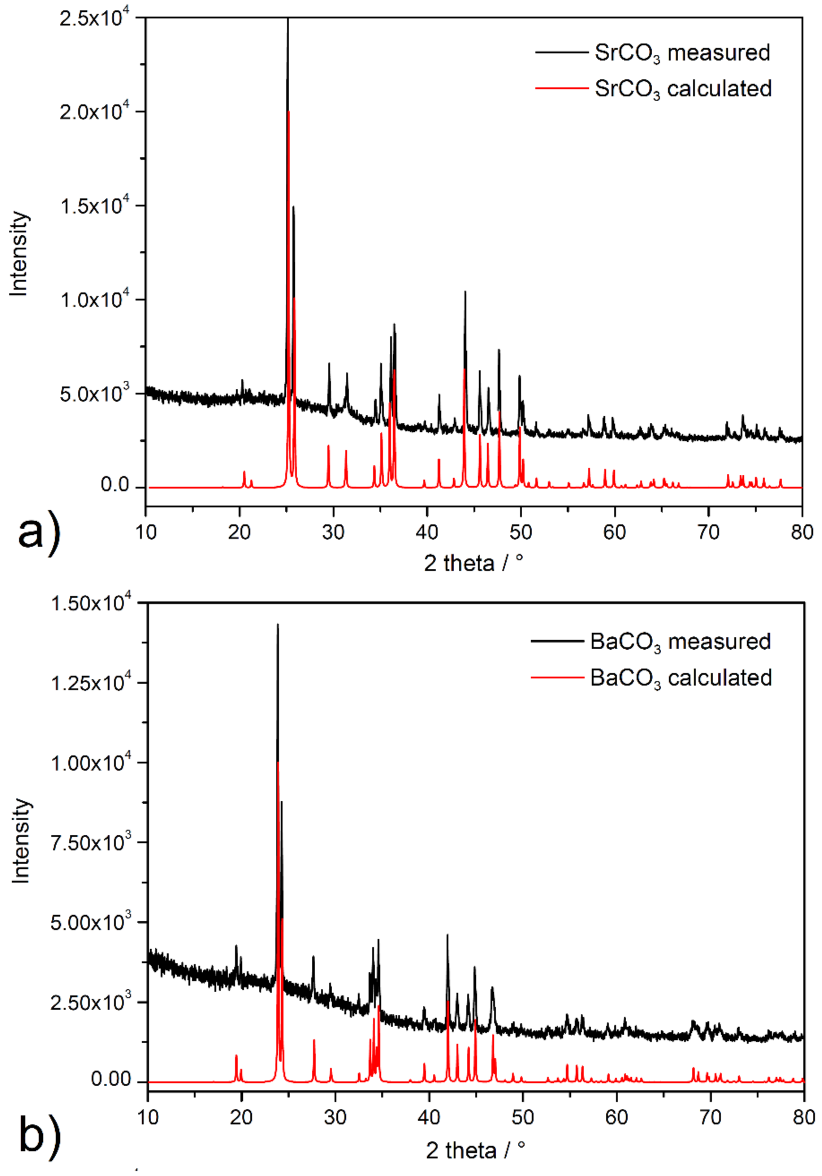

Figure S1 Measured PXRD patterns of final decomposition products and comparison with calculated patterns of $SrCO_3$ and $BaCO_3$.
